# The N-acetylglucosaminyltransferase Radical fringe contributes to defects in JAG1-dependent turnover and signaling of NOTCH3 CADASIL mutants

**DOI:** 10.1016/j.jbc.2024.107787

**Published:** 2024-09-19

**Authors:** Shodai Suzuki, Taiki Mashiko, Yohei Tsukamoto, Miyu Oya, Yuki Kotani, Saki Okawara, Takemi Matsumoto, Yuki Mizue, Hideyuki Takeuchi, Tetsuya Okajima, Motoyuki Itoh

**Affiliations:** 1 Department of Biochemistry, Graduate School of Pharmaceutical Sciences, Chiba University, Chiba, Chiba, Japan; 2 Department of Molecular Biochemistry, Graduate School of Medicine, Nagoya University, Nagoya, Aichi, Japan; 3 Department of Biochemistry, School of Pharmaceutical Sciences, University of Shizuoka, Shizuoka, Shizuoka, Japan; 4 Institute for Glyco-core Research (iGCORE), Graduate School of Medicine, Nagoya University, Nagoya, Aichi, Japan; 5 Research Institute of Disaster Medicine, Chiba University, Chiba, Chiba, Japan; 6 Health and Disease Omics Center, Chiba University, Chiba, Chiba, Japan

**Keywords:** notch pathway, protein degradation, glycosylation, aging, vascular biology, CADASIL, NOTCH3, fringe, pericyte

## Abstract

Cerebral autosomal dominant arteriopathy with subcortical infarcts and leukoencephalopathy (CADASIL) is a genetic vascular dementia characterized by age-related degeneration of vascular mural cells and accumulation of a NOTCH3 mutant protein. NOTCH3 functions as a signaling receptor, activating downstream gene expression in response to ligands like JAG1 and DLL4, which regulate the development and survival of mural cells. This signal transduction process is thought to be connected with NOTCH3 endocytic degradation. However, the specific cellular circumstances that modulate turnover and signaling efficacy of NOTCH3 mutant protein remain largely unknown. Here, we found elevated *NOTCH3* and Radical fringe *(RFNG)* expression in senescent human pericyte cells. We then investigated impacts of RFNG on glycosylation, degradation, and signal activity of three NOTCH3 CADASIL mutants (R90C, R141C, and C185R) in EGF-like repeat-2, 3, and 4, respectively. Liquid chromatography with tandem mass spectrometry analysis showed that RFNG modified NOTCH3 WT and C185R to different degrees. Additionally, coculture experiments demonstrated that RFNG significantly promoted JAG1-dependent degradation of NOTCH3 WT but not that of R141C and C185R mutants. Furthermore, RFNG exhibited a greater inhibitory effect on JAG1-mediated activity of NOTCH3 R141C and C185R compared to that of NOTCH3 WT and R90C. In summary, our findings suggest that NOTCH3 R141C and C185R mutant proteins are relatively susceptible to accumulation and signaling impairment under cellular conditions of RFNG and JAG1 coexistence.

Cerebral autosomal dominant arteriopathy with subcortical infarcts and leukoencephalopathy (CADASIL) is a cerebral small vessel disease characterized by age-dependent symptoms, ranging from migraine with aura to ischemic attacks and dementia (1). The genetic factor responsible for this disease has been identified as a point mutation in *NOTCH3*, mainly expressed in vascular smooth muscle cells and pericytes (2). The pathogenesis is featured with the degeneration of mural cells and deposits of granular osmiophilic materials (GOMs); this protein aggregate appears in the extracellular space adjacent to mural cells, containing the ectodomain of the NOTCH3 (N3ECD) mutant protein (2, 3, 4, 5, 6, 7, 8, 9, 10). Additionally, aging increases the NOTCH3 aggregation and the loss of mural cells in a mouse model (11, 12), leading to the hypothesis that changes in the cellular environment with aging may contribute to the accumulation of N3ECD mutants and the pathogenesis of CADASIL. However, the specific cellular conditions that contribute to the accumulation of N3ECD mutants have not been identified.

The NOTCH receptor is a transmembrane protein with two domains: the NOTCH extracellular domain (NECD) and the NOTCH intracellular domain (NICD) (13). The Notch signaling pathway is initiated by the binding of the NECD to Notch ligands, such as Delta-like 1, 3, and 4 (DLL1, 3, and 4) and Jagged 1 and 2 (JAG1, and 2) (14). The ligands physically dissociate the NECD through a process known as transendocytosis, followed by lysosomal degradation (15, 16, 17). Consequently, the NOTCH intracellular domain is released into the nucleus to activate downstream target gene expression (14).

The N3ECD contains 34 EGF repeats (EGFrs), each of which has three disulfide bonds with six cysteines (18, 19). Most CADASIL-associated point mutations result in an odd number of cysteine residues within an EGFr of the N3ECD (19). High-risk CADASIL mutations often occur in NOTCH3 EGFr1-6 (amino acids 40–272), outside the ligand-binding domain (EGFr10 and 11) (19, 20, 21). Pathogenesis of CADAIL is attributed to neomorphic toxic effects of NOTCH3 CADASIL mutant proteins. In accordance with this, previous studies have shown spontaneous multimerization in NOTCH3 mutants, including C49Y, R90C, R75P (22), R133C (23, 24, 25), R141C (22), and C183R (23, 24). Some NOTCH3 mutants in EGFr1-3 are reported to exhibit structural alterations compared to NOTCH3 WT (22, 26). However, our recent study using a coculture system demonstrated that NOTCH3 C49Y, R90C, R141C, and C185R expressed in human embryonic kidney (HEK) 293 cells are degraded by JAG1 as efficiently as NOTCH3 WT (27). On the other hand, signaling defects in NOTCH3 mutant proteins also contribute to mural cell degeneration in CADASIL. This hypothesis is supported by previous research using NOTCH3 KO animals, which exhibit vascular smooth muscle cell (VSMC) apoptosis (28, 29, 30), reduced proliferating pericytes (31), brain hypoperfusion, and glymphatic system deficiency (32). VSMCs with NOTCH3 R133C mutation show diminished upregulation of NOTCH3 downstream genes, *PDGFRβ*, *HES1*, and *HEY1*, in response to JAG1 than those from control subjects (33). Conversely, previous studies indicate that JAG1 activates NOTCH3 R90C and C212S in NIH-3T3 fibroblast cells (34), C185R in HEK293 cells (35), and R133C and C183R in A7r5 cells, rat aortic smooth muscle cells (36), to a similar extent as NOTCH3 WT. Accordingly, cellular environmental factors, beyond pathogenic mutations, may alter turnover and signaling efficacy of NOTCH3 mutant proteins. However, other factors influencing NOTCH3 mutant protein degradation and activity remain unclear.

One such modifiable factor is *O-*glycosylation of NOTCH3. *O-*fucose is attached to the consensus sequence (C^2^ -X-X-X-X-S/T-C^3^) of an EGFr by *O-*Fut1 and further elongated with *N-*acetylglucosamine (GlcNAc) by Fringe, galactose by Gal-T, and sialic acid by Sia-T (37). Fringe, including Lunatic fringe (LFNG), Manic fringe (MFNG), and Radical fringe (RFNG), is known to modulate NOTCH signaling (37, 38, 39, 40, 41). LFNG and MFNG enhance DLL1-stimulated NOTCH1 signal activity but reduce JAG1-stimulated signaling; in contrast, RFNG enhances both (39). In another study, MFNG also promotes the degradation of NOTCH3 WT in HEK293 cells (42). Under these circumstances, the effects of Fringe on the degradation and signaling activity of NOTCH3 CADASIL mutant proteins during ligand interaction remain to be elucidated.

Additionally, it is crucial to consider the NOTCH ligands that interact with the NOTCH3 CADASIL mutant proteins. NOTCH1 and NOTCH2 exhibit distinct affinities and activations in response to various ligands (40). Previous *in vivo* studies have shown that endothelial cell-specific JAG1 plays a role in the proper development and function of vascular mural cells through NOTCH3 signaling in mouse embryos (43) and retinas (29, 44). *In vitro* studies have suggested that NOTCH3 in vascular mural cells is activated by endothelial-specific JAG1 (45) and recombinant JAG1 protein (46). Moreover, DLL4 has been implicated in angiogenesis through NOTCH1 signaling in previous studies using the mouse retina (44, 47). Schulz *et al.* revealed that DLL4 induces NOTCH3 signaling in pericytes (48). However, the ligands responsible for turnover and signaling activity of the NOTCH3 CADASIL mutant are not yet fully understood.

In this study, we investigated the impact of fringe on the glycosylation, proteostasis, and signaling activity of NOTCH3 mutant proteins—R90C, R141C, and C185R, which correspond to CADASIL-associated mutations in EGFr2, 3, and 4, respectively. Our findings revealed upregulation of RFNG and NOTCH3 in a senescent pericyte cell line. Through LC–MS/MS analysis of purified NOTCH3 EGFr1-12 proteins, we observed that RFNG modified NOTCH3 WT and C185R to varying extents. Furthermore, our coculture assay demonstrated that RFNG promoted JAG1-dependent degradation of NOTCH3 WT but not that of NOTCH3 R141C and C185R. Additionally, RFNG was more effective at inhibiting the JAG1-dependent signaling activity of NOTCH3 R141C and C185R compared to that of NOTCH3 WT and R90C. Overall, NOTCH3 R141C and C185R mutants exhibited greater susceptibility to protein accumulation and impaired signaling than NOTCH3 WT when RFNG and JAG1 were present in cellular conditions.

## Results

### Expression patterns of Notch3, Fringe, and Notch ligands in murine brain vasculature

We investigated which Fringe family genes are coexpressed with NOTCH3 in the brain vasculature using a single-cell RNA-seq database of vascular cells from WT adult young mouse brains (http://betsholtzlab.org/VascularSingleCells/database.html) (49, 50). *Notch3* expression was highly restricted to mural cells, such as pericytes and smooth muscle cells (SMCs) (Fig. S1*A*). Mural cells also expressed *Notch2*, but the average counts were lower than those of *Notch3* (Fig. S1, *A* and *B*). Neither *Lfng* nor *Mfng* was expressed in the mural cells (Fig. S1, *C* and *D*). However, the mural cells did express *Rfng* (Fig. S1*E*). Therefore, *Notch3* and *Rfng* are coexpressed in mural cells, suggesting that RFNG may play an important role in the posttranslational glycosylation of NOTCH3 in the cells of cerebrovascular system. We next explored the expression of the following Notch ligands: Dll1, 3, 4, and Jag1 and Jag2. *Dll1* and *Dll3* were barely expressed in any vascular cell type (Fig. S1, *F* and *G*), but *Dll4* was restricted to endothelial cells (Fig. S1*H*). In contrast, *Jag1* was broadly expressed in pericytes, SMCs, fibroblasts, endothelial cells, and astrocytes (Fig. S1*I*), but *Jag2* was found in only endothelial cells (Fig. S1*J*). Hence, JAG1, JAG2, and DLL4 may regulate NOTCH3 signaling activity and turnover in the brain vasculature.

### Long-term culture-induced senescence increases RFNG expression in a human pericyte cell line

**Figure 1:**
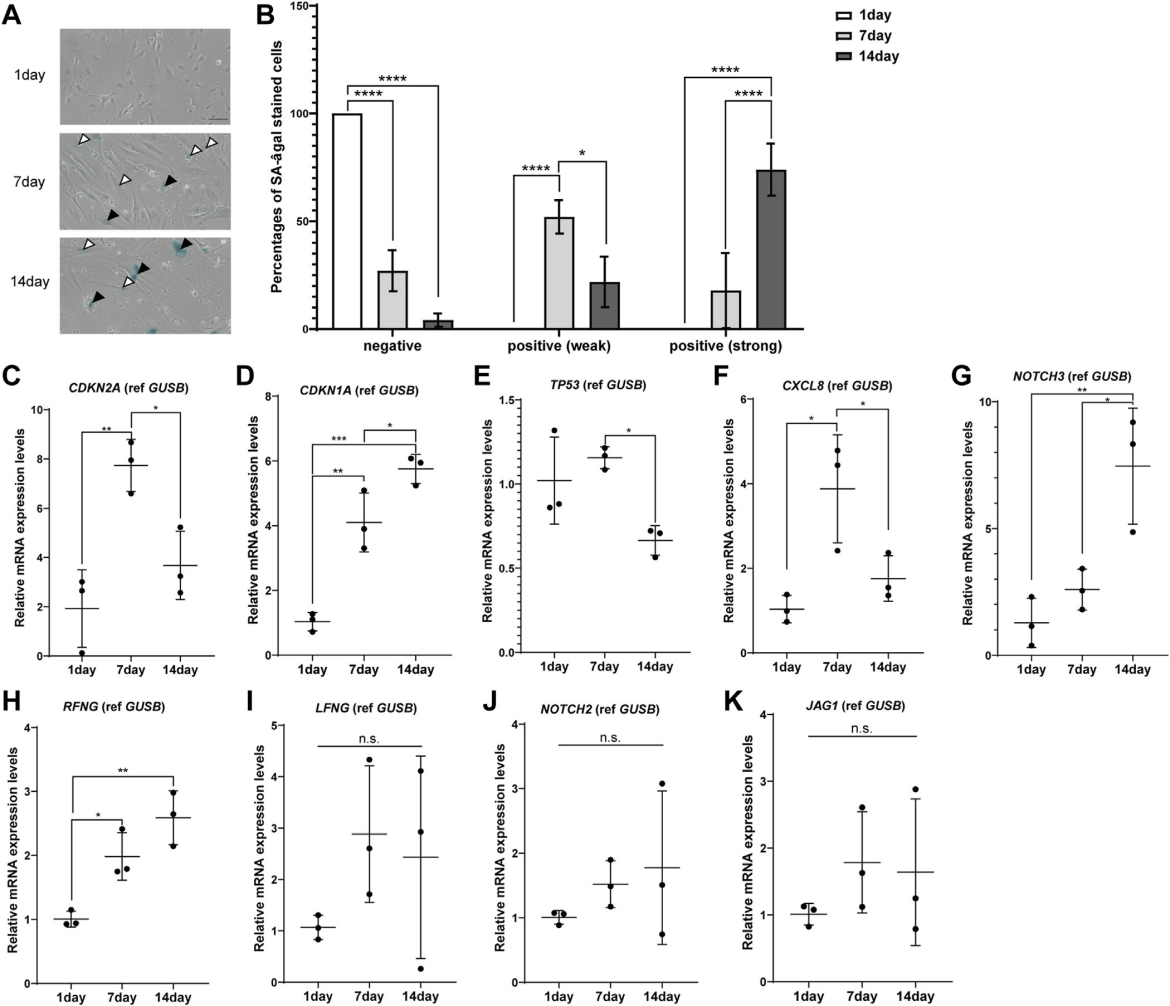
**Long-term culture-induced senescence enhances the expression of NOTCH3 and RFNG in a human brain pericyte cell line.***A*, representative images of senescence-associated β-galactosidase (SA-β-gal)-stained HBPC/ci37 cells after culture for 1, 7, or 14 days. The scale bar represents 10 μm. *B*, percentages of negative and positive (weak and strong) SA-β-gal-stained cells relative to the total number of cells (independent biological replicates N = 3; total cell number, 1 day: n = 329, 7 days: n = 261, 14 days: n = 224). *C-K*, qRT‒PCR showing the relative mRNA levels of *CDKN2A* (C), *CDKN1A* (D), *TP53* (E)*, CXCL8* (F), *NOTCH3* (G), *RFNG* (H), *LFNG* (I), *NOTCH2* (J), and *JAG1* (K) in HBPC/ci37 cells cultured for 1, 7, and 14 days (independent biological replicates N = 3). *GUSB* was used as a reference gene. Data information: In *B*-*K*, data are presented as mean ± SDs. n.s., not significant, ∗*p* < 0.05, ∗∗*p* < 0.01, ∗∗∗*p* < 0.001 (B: Tukey’s *post hoc* test following two-way ANOVA; C-K: Tukey’s *post hoc* test following one-way ANOVA). JAG1, Jagged 1; LFNG, Lunatic fringe; qRT-PCR, quantitative reverse transcription PCR; RFNG, Radical fringe.

Aging is a critical factor for the accumulation of the NOTCH3 mutant protein and pericyte loss in CADASIL (11, 12). Previous studies have shown that pericyte cellular senescence, characterized with upregulation of senescence-associated β-galactosidase (SA-β-gal), is a hallmark of vascular aging (51, 52). Furthermore, previous studies have shown that NOTCH3 expression is induced in senescent cells during long-term cultures (53, 54). We next investigated whether aging regulates the expression of NOTCH3, NOTCH2, Fringe, and JAG1 using the human brain pericyte cell line HBPC/ci37 (55). Transduced temperature-sensitive SV40T promotes HBPC/ci37 cell proliferation at 33 °C but becomes inactivated, leading to cell differentiation at 37 °C (55). Long-term culture is known to induce senescence in cultured cells. Senescent cells exhibit morphological changes, such as a flattened cell shape, enlarged cell body and nucleus; and upregulation of senescence-related genes, such as SA-β-gal, CDKN2A, CDKN1A, TP53, and CXCL8 (56). We cultured HBPC/ci37 cells at 37 °C for up to 14 days in a serum-depleted medium to induce senescence. Following this period, we examined the cell morphology and expression of senescence-related genes. HBPC/ci37 cells cultured for 7 or 14 days exhibited a more flattened shape and more enlarged cell size and nuclei than did those cultured for a day (Fig. 1*A*). SA-β-gal activity was higher in the cells after 7 and 14 days than after 1 day (Fig. 1, *A* and *B*). Quantitative PCR of senescence markers was performed with PUM1 and GUSB as reference genes, because their expression remains stable during *in vitro* aging (57). Culture for 7 days, but not for 14 days, led to elevated mRNA expression of *CDKN2A* (Figs. 1*C* and S2*A*). Culture for 7 or 14 days increased *CDKN1A* mRNA expression in a time-dependent manner (Figs. 1*D* and S2*B*). However, long-term culture reduced the *TP53* mRNA level (Figs. 1*E* and S2*C*). CXCL8 was also increased by culture for 7 days but not for 14 days (Figs. 1*F* and S2*D*). Taken together, the results showed that culture for 7 to 14 days could partially induce HBPC/ci37 cell senescence. HBPC/ci37 cells expressed *RFNG* at a level similar to that of *NOTCH3* but higher than that of *LFNG* and *MFNG* (Fig. S2*E*), consistent with the mouse single-cell transcriptome (Fig. S1, *A* and *C*–*E*). Senescence enhanced *NOTCH3* and *RFNG* (Figs. 1, *G*, *H* and S2, *F* and *G*) but not *NOTCH2, LFNG,* or *JAG1* (Figs. 1, *I*, *K* and S2, *H*–*J*). Meanwhile, the 14-day culture did not increase the number of apoptotic cells stained by propidium iodide (PI) (Fig. S2*K*). These results suggest that senescence may increase the amount of NOTCH3 and its glycosylation by RFNG in pericytes.

### RFNG elongates the *O-*fucose glycan on EGFr11 of NOTCH3 C185R more than on EGFr4

**Figure 2:**
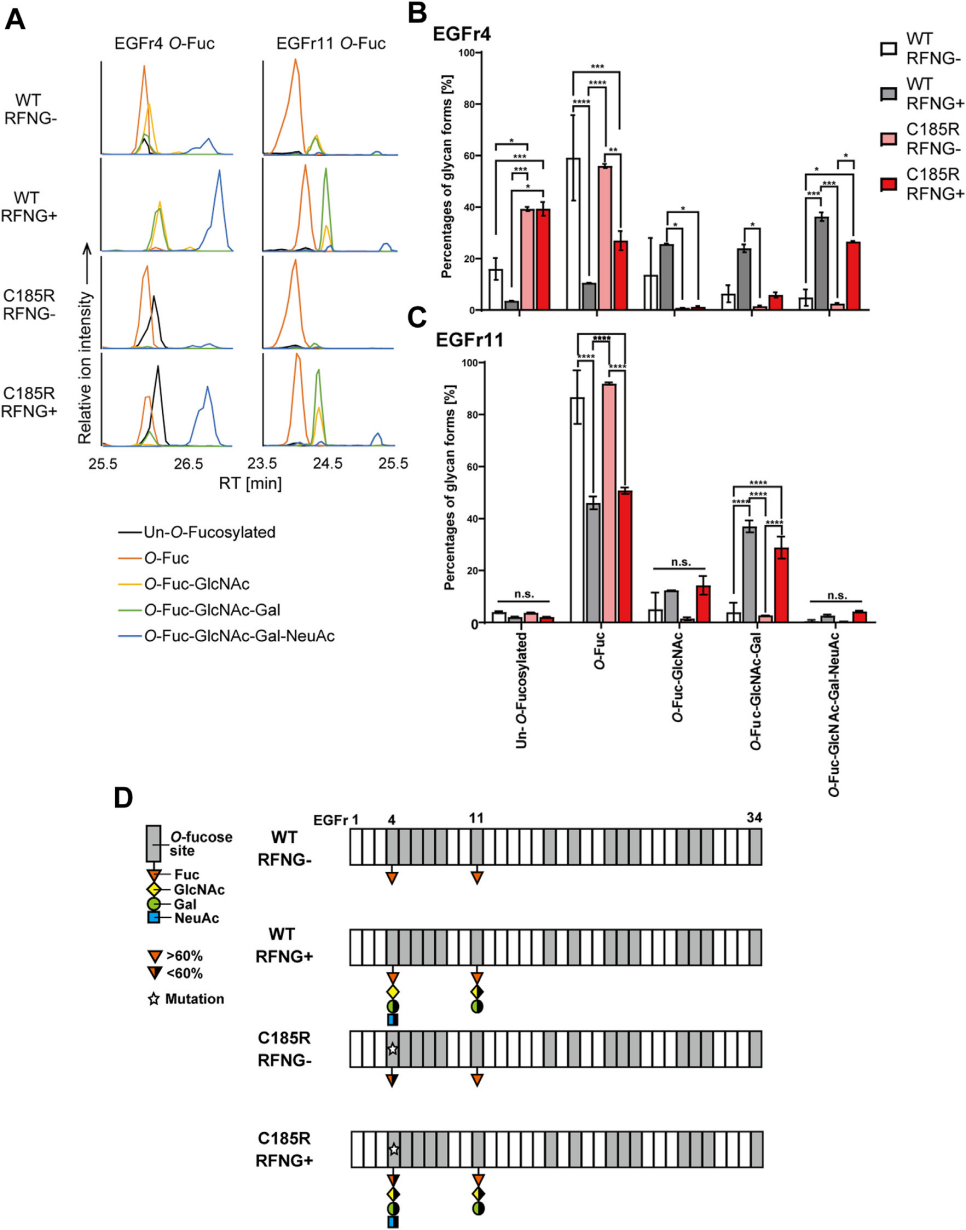
**Analysis of RFNG-mediated modification of *O-*fucose glycan in NOTCH3 WT and C185R.***A*, extracted ion chromatograms (EIC) of trypsin digests containing O-fucose sites at NOTCH3 EGFr4 and 11, as determined by LC‒MS/MS using purified NOTCH3 EGFr1-12 WT and C185R, with or without RFNG. Each line graph represents the peptides unmodified with *O*-fucose glycan (Un-*O-*fucosylated, *black*) or modified with *O-*Fuc (*orange*), *O-*Fuc-GlcNAc (*yellow*), *O-*Fuc-GlcNAc-Gal (*green*), or *O-*Fuc-GlcNAc-Gal-NeuAc (*blue*). Since the EGFr4 peptide also had mono-*O-*glucose modification by POGLUT2/3 at ∼90% in addition to *O-*fucose, the presented EIC included *O-*glucose modification. *(B*, *C)* Proportion of *O-*fucose glycan forms of EGFr4 *(B)* and EGFr11 *(C)* in the NOTCH3 WT and C185R with or without RFNG. *Bars* show the relative peak heights of peptides unmodified with *O*-fucose glycan (Un-*O-*fucosylated), *O-*fucose glycan modified with *O-*Fuc, *O-*Fuc-GlcNAc, *O-*Fuc-GlcNAc-Gal, and *O-*Fuc-GlcNAc-Gal-NeuAc (independent biological replicates N = 2). *D*, a summary of the *O-*fucose glycan states in NOTCH3 WT and C185R with or without RFNG. Data information: In *B*-*C*, data are presented as mean ± SDs. n.s., not significant, ∗*p* < 0.05, ∗∗*p* < 0.01, ∗∗∗*p* < 0.001, ∗∗∗∗*p* < 0.0001 (Tukey’s *post hoc* test following three-way ANOVA). EGFr, EGF repeat; GlcNAc, *N-*acetylglucosamine; LC-MS/MS, liquid chromatography with tandem mass spectrometry; RFNG, Radical fringe.

The results of our study led us to hypothesize that RFNG plays a critical role in regulating the glycosylation and turnover of the NOTCH3 CADASIL mutant protein. A previous report demonstrated that the fragmented proteins of mouse NOTCH3 CADASIL-like mutants, such as R91C, R170C, and C213S, exhibited reduced elongation of *O-*fucose by LFNG (58). To examine whether RFNG extends *O-*fucose from the human NOTCH3 WT and C185R, which is a CADASIL mutant in EGFr4, we used liquid chromatography with tandem mass spectrometry (LC-MS/MS); this method was used to determine the O-fucose glycan states in NOTCH1 and NOTCH2 in previous studies (39, 40). Detecting the *O-*glycan state of our pericyte cell line *via* LC-MS/MS posed a challenge due to the low transfection efficiency. Therefore, we used a HEK293 cell line, which is suitable for production of NOTCH proteins (59, 60). We produced the NOTCH3 EGFr1-12 WT and C185R-FcHis fragment proteins from the HEK293 cell line overexpressing RFNG, whose expression was induced by the addition of doxycycline (61). Among the *O-*fucose consensus sites in NOTCH3 EGFr1-12 (58), those in EGFr4 and 11 were analyzed, because the C185R mutation reduces the cysteine in EGFr4, and the ligand-binding domain includes EGFr11. The purified NOTCH3 fragments with or without RFNG were digested with trypsin and subjected to LC-MS/MS. We subsequently searched for *O-*fucose glycans in the peptides corresponding to EGFr4 and 11 (Fig. 2*A*). The quantification of the relative peak heights revealed the percentages of the *O-*fucose glycan forms: no glycan, *O-*Fuc, *O-*Fuc-GlcNAc, *O-*Fuc-GlcNAc-Gal, or *O-*Fuc-GlcNAc-Gal-NeuAc (Fig. 2, *B* and *C*). RFNG efficiently elongated the *O-*fucose of EGFr4 in the NOTCH3 WT (Fig. 2*B*). Although the O-fucose glycan in the NOTCH3 C185R mutant was reduced overall compared to that in the WT in the EGFr4, RFNG extended approximately half of the *O-*fucose content (Fig. 2*B*). In contrast, the total content of *O-*fucose glycans and the RFNG-mediated extension were comparable between the WT and C185R in the EGFr11 (Fig. 2*C*). Therefore, RFNG partially elongated the O-fucose of NOTCH3 C185R in EGFr4 but similarly did so in EGFr11 compared to NOTCH3 WT (Fig. 2*D*).

### RFNG alters the clustering size and/or structure of the NOTCH3 EGFr1-12 C185R

**Figure 3:**
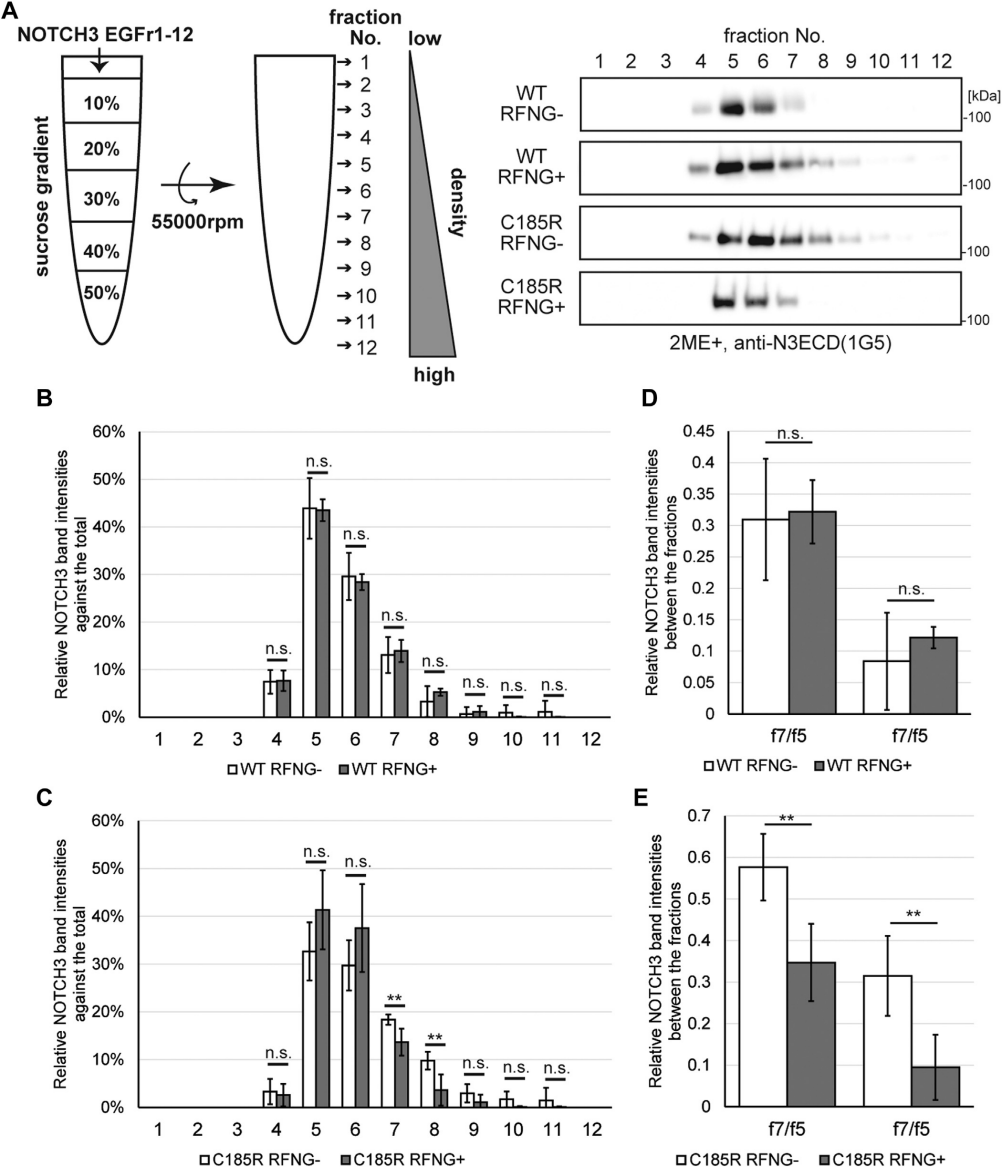
**RFNG regulates the clustering size and structure of the NOTCH3 EGFr1-12 C185R mutant.***A*, representative blot of a sucrose density gradient ultracentrifugation assay using purified NOTCH3 EGFr1-12 WT and C185R with or without RFNG-mediated glycosylation. *B* and *C,* percentages of NOTCH3 EGFr1-12 WT (B) and C185R (C) in fractions against the total amount of all fractions, as determined by the Western blot shown in (A) (independent biological replicates N = 5). *D* and *E*, relative band intensities of NOTCH3 WT *(D)* and C185R *(E)* in fractions 7 and 8 compared to those in fraction 5 (independent biological replicates N = 5). Data information: In *B*-*D*, data are presented as mean ± SDs. ∗∗*p* < 0.01, n.s., not significant (Unpaired two-tailed Student’s *t* test). EGFr, EGF repeat; RFNG, Radical fringe.

Previous studies have shown the spontaneous multimerization of the NOTCH3 CADASIL mutant fragments (23, 24) and abnormal disulfide bond pairs in an intramolecular way (22, 26). Moreover, NOTCH1 undergoes clustering before signal activation by JAG1 (15). To investigate the impact of RFNG-mediated modification of *O-*fucose on the disulfide bond-based multimerization of NOTCH3 WT and C185R, we subjected the NOTCH3 EGFr1-12 WT and C185R Fc-fusion proteins to reducing and nonreducing SDS‒PAGE. In reducing SDS‒PAGE, the NOTCH3 EGFr1-12 WT and C185R were observed to be monomers with a single band at ∼110 kDa (Fig. S3*A*). The molecular weights of these proteins increased due to the *O-*fucose modification of NOTCH3 by RFNG (Fig. S3*A*). In contrast, nonreducing SDS‒PAGE revealed multiple bands corresponding to NOTCH3 WT and C185R (Fig. S3*B*). NOTCH3 WT was detected by a major band at ∼200 kDa, which might be the dimerized form through the Fc region (Fig. S3*B*). NOTCH3 C185R also exhibited a dimer with mobility similar to that of the WT (dimer 1) and an additional band lower than 180 kDa (dimer 2, Fig. S3*B*). This difference in mobility between the WT and C185R might reflect intramolecular abnormal disulfide bond pairs in the NOTCH3 C185R compared to the WT, as suggested by a previous report (22). In addition, the multimers of NOTCH3 C185R were increased compared to those of NOTCH3 WT (Fig. S3*B*), consistent with previous reports (23, 24). We then quantified the proportion of the dimers and multimers among the total proteins in the NOTCH3 WT and C185R (Fig. S3, *C* and *D*). RFNG reduced the multimers of the NOTCH3 WT and C185R (Fig. S3, *C* and *D*). Thus, RFNG ameliorated the disulfide bond-based multimerization of the NOTCH3 EGFr1-12 WT and C185R. We also evaluated the effect of RFNG on the clustering size and structure of NOTCH3 WT and C185R by sucrose density gradient ultracentrifugation, which was used to determine the sizes and structures of amyloid-β, prion protein, and α-synuclein in previous studies (62, 63, 64). The purified NOTCH3 EGFr1-12 WT and C185R proteins were fractionated into 12 fractions according to density by ultracentrifugation after loading into sucrose gradient solutions ranging from 10% to 50% (Fig. 3*A*). The amounts of NOTCH3 WT and C185R were quantified *via* Western blotting and calculated relative to the total (Fig. 3, *B* and *C*). When RFNG was not induced, NOTCH3 C185R was detected in fractions with higher density than NOTCH3 WT (Fig. 3, *B* and *C*). This difference might be partially explained by abnormal disulfide bonds and increased multimerization, as indicated by the nonreducing SDS‒PAGE results (Fig. S3*B*). Furthermore, RFNG did not alter the distribution of NOTCH3 WT (Fig. 3*B*); however, it shifted that of NOTCH3 C185R to fractions with lower density (Fig. 3*C*), as shown by the relative amounts of NOTCH3 WT and C185R in fractions 7 and 8 compared with those in fraction 5 (Fig. 3, *D* and *E*). These data indicated that RFNG reduced the clustering size and/or changed the structure of NOTCH3 EGFr1-12 C185R but not that of the WT.

### RFNG does not affect JAG1-mediated degradation of the soluble preclustered NOTCH3 C185R protein

**Figure 4:**
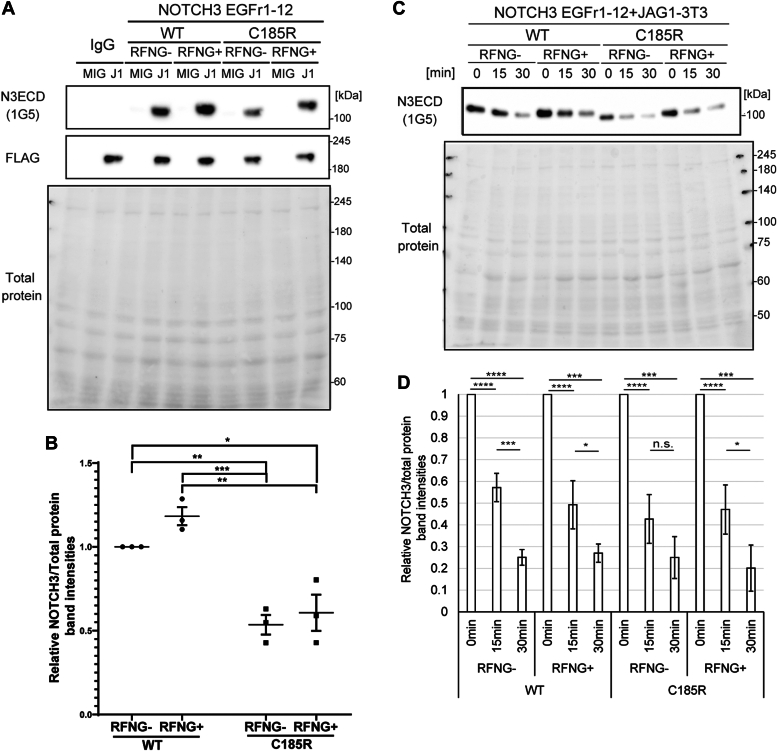
**RFNG does not affect JAG1-mediated degradation of preclustered NOTCH3 WT or C185R soluble proteins.***A*, representative Western blot of lysates collected from MIG-3T3 and JAG1-3T3 cells after binding to purified NOTCH3 EGFr1-12 WT or C185R, with or without RFNG. NOTCH3 and JAG1 were immunoblotted with antibodies against NOTCH3ECD and FLAG. Ponceau stained total protein. *B*, relative amount of NOTCH3 EGFr1-12 WT and C185R binding to JAG1, as determined by the blots shown in (A) (independent biological replicates N = 3). The values were calculated by normalizing the relative NOTCH3 band intensities to those of the NOTCH3 WT without RFNG (WT RFNG-). *C*, representative blot of lysates collected from JAG1-3T3 cells after taking up purified NOTCH3 EGFr1-12 WT and C185R, with or without RFNG-mediated modification. *D*, relative amounts of NOTCH3 EGFr1-12 WT and C185R after degradation by JAG1, as determined by the blots shown in (C) (independent biological replicates N = 3). The NOTCH3 band intensities were divided by the total protein band intensities. The values are shown as NOTCH3 band intensities relative to those at 0 min. Data information: In *B* and *D*, data are presented as mean ± SDs. ∗*p* < 0.05, ∗∗*p* < 0.01, ∗∗∗*p* < 0.001 (B: Tukey’s *post hoc* test following two-way ANOVA; C: Tukey’s *post hoc* test following one-way ANOVA). EGFr, EGF repeat; JAG1, Jagged 1; RFNG, Radical fringe.

Given the findings of a previous study in which the JAG1-mediated endocytosis of NOTCH1 requires clustering (15), RFNG may impair the JAG1-dependent turnover of the NOTCH3 EGFr1-12 C185R by affecting the clustering size and structure. To examine whether RFNG influences the binding and degradation of NOTCH3 EGFr1-12 WT and C185R by JAG1, the NOTCH3 EGFr1-12 WT and C185R Fc-fusion proteins were clustered with anti-Fc and incubated on ice with the NIH-3T3 cell line overexpressing JAG1 and GFP (JAG1-3T3) or that expressing only GFP (MIG-3T3) (65). After unbound NOTCH3 fragments were removed, the cell lysates were subjected to Western blotting with an anti-N3ECD antibody (Fig. 4, *A* and *B*). Although the NOTCH3 EGFr1-12 C185R was found to bind less strongly to JAG1 than was the WT (Fig. 4*B*), RFNG-mediated modification did not change the affinity of these proteins (Fig. 4*B*). To verify the degradation of NOTCH3 EGFr1-12, JAG1-3T3 cells were incubated at 37 °C after binding to the NOTCH3 fragments, thereby inducing endocytosis (Fig. 4, *C* and *D*). JAG1 markedly reduced the NOTCH3 WT and C185R within 15 and 30 min. However, RFNG did not affect the degradation of these NOTCH3 proteins (Fig. 4, *C* and *D*). Altogether, whereas the C185R mutation impeded the binding of NOTCH3 EGFr1-12 to JAG1, RFNG did not affect JAG1-mediated endocytosis or degradation of the preclustered NOTCH3 EGFr1-12 WT or C185R protein.

### RFNG does not change the ligand-independent degradation of full-length NOTCH3 WT or C185R

**Figure 5:**
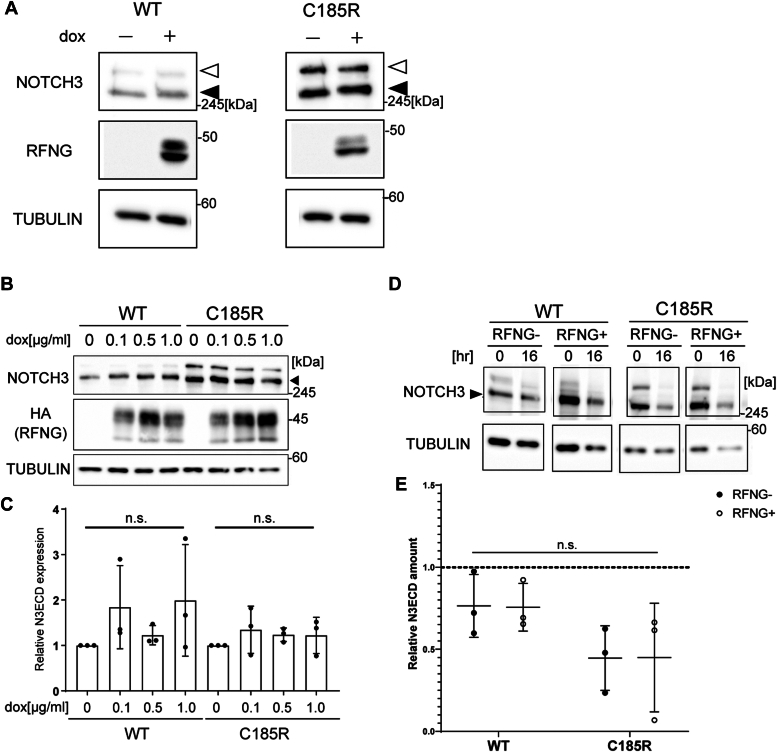
**RFNG does not alter the steady-state levels of the NOTCH3 WT or C185R protein.***A*, representative Western blot of lysates from cultured N3WT/C185R-RF-HeLa cells with and without doxycycline-inducible expression of RFNG. NOTCH3 and RFNG were detected by antibodies against human NOTCH3 extracellular domain (ECD) and human RFNG. TUBULIN was blotted with an antibody against human/mouse α-tubulin as a loading control. The *white* and *black arrowheads* indicate full-length NOTCH3 and the ECD, respectively. *B*, representative Western blot of lysates from cultured N3WT/C185R-RF-HeLa cells treated with or without 0, 0.1, 0.5, or 1 μg/ml doxycycline. RFNG was detected by an antibody against HA, which tagged RFNG. The *black arrowheads* indicate N3ECDs. *C*, relative amounts of N3ECD WT and C185R with or without RFNG expression, as determined by Western blotting (B) (independent biological replicates N = 3). N3ECD band intensities were normalized to those observed without doxycycline. *D*, representative Western blot of cell lysates collected from cultured N3WT-RF-HeLa and N3C185R-RF-HeLa cells with or without RFNG expression and treated with puromycin for 0 or 16 h. The *black arrowheads* indicate N3ECDs. *E*, relative band intensities of N3ECD at 16 h normalized to those observed at 0 h, as determined by Western blotting in (D) (independent biological replicates N = 3). Data information: In *C* and *E*, data are presented as mean ± SD. n.s., not significant (C: Tukey’s *post hoc* test following one-way ANOVA; E: Tukey’s *post hoc* test following two-way ANOVA). HA, hemagglutinin; N3ECD, NOTCH3 extracellular domain; RFNG, Radical fringe.

Given that we did not observe an effect of RFNG on the turnover of the soluble NOTCH3 WT or C185R proteins, we next investigated the impact of RFNG on the degradation of the full-length NOTCH3 WT and C185R expressed in cells by generating stable HeLa cell lines coexpressing full-length NOTCH3 WT or C185R and RFNG (hereafter N3WT/C185R-RF-HeLa cells). In these cell lines, NOTCH3 WT or C185R was expressed constitutively, and the expression of RFNG was controlled by the addition of doxycycline (61). First, the protein expression of the NOTCH3 extracellular domain (N3ECD) was examined *via* Western blotting with an anti-N3ECD antibody. The addition of dox to the culture medium induced RFNG expression and slightly increased the molecular mass of both the N3ECD WT and C185R (Fig. 5*A*). The protein levels of both N3ECD WT and C185R did not change dramatically upon dox treatment (Fig. 5, *B* and *C*). Thus, RFNG modified both the N3ECD WT and C185R but did not regulate their steady-state protein levels. The effect of RFNG on the ligand-independent degradation of N3ECD WT and C185R was investigated using puromycin, a protein synthesis inhibitor. After culturing N3WT-RF-HeLa and N3C185R-RF-HeLa cells for 24 h in the presence or absence of dox, the cells were treated with puromycin. Treatment with puromycin for 16 h did not increase the apoptosis of adherent cells, as not stained by PI (Fig. S4), and thus, the whole cell lysates were collected at this time point and 0 h. Thereafter, the lysates collected at 0 h and 16 h were subjected to Western blotting with an anti-N3ECD antibody and calculated relative NOTCH3 amount at 16 h to that at 0 h (Fig. 5, *D* and *E*). Within 16 h, approximately 75% of the N3ECD WT was degraded, regardless of RFNG expression (Fig. 5*E*). Similarly, N3ECD C185R was reduced to approximately 50%, which was not affected by RFNG (Fig. 5*E*). Thus, RFNG did not influence the ligand-independent turnover of NOTCH3 WT or C185R in cells.

### RFNG promotes JAG1-dependent degradation of NOTCH3 WT but not that of R141C and C185R

**Figure 6:**
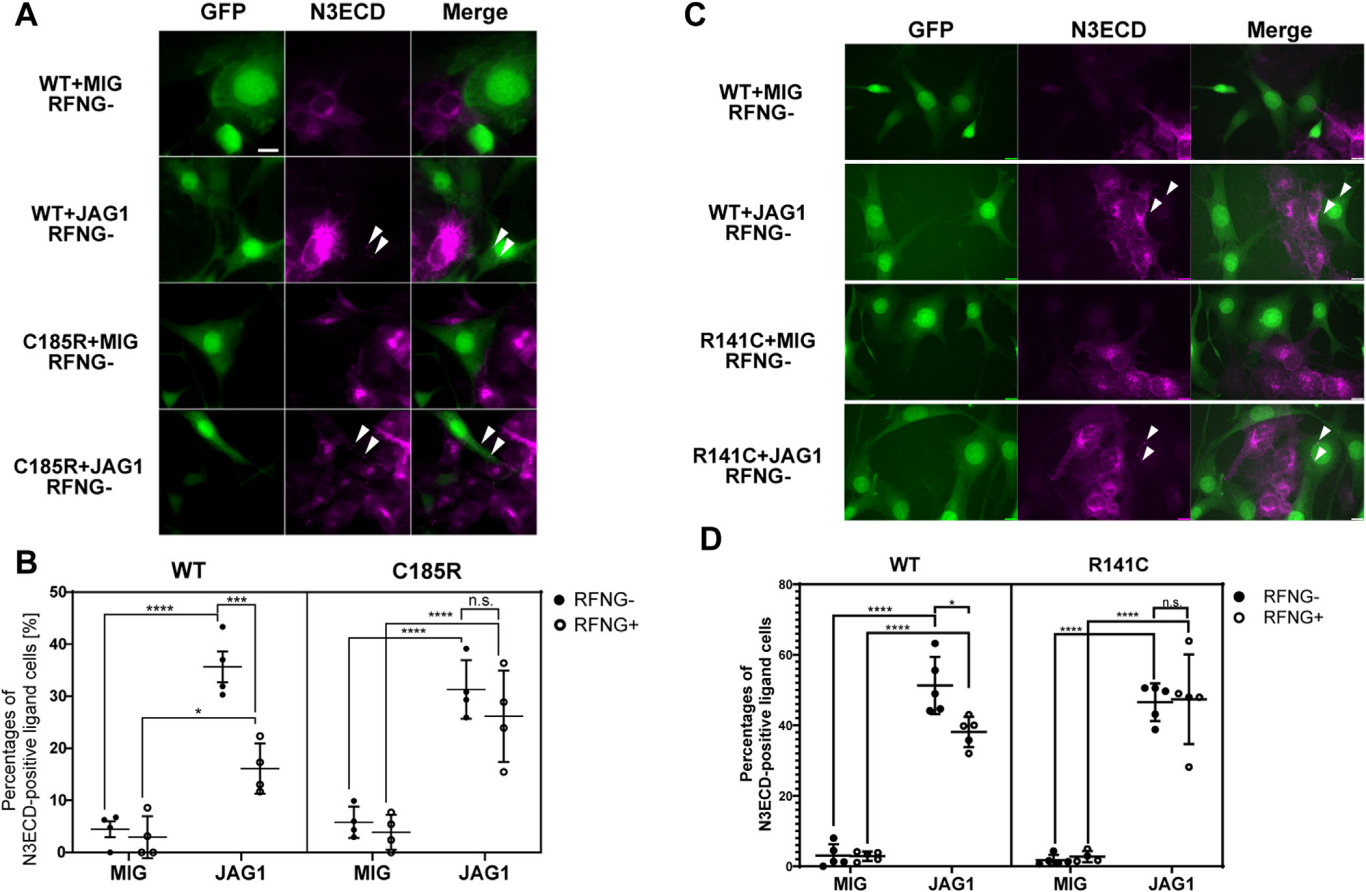
**RFNG enhances JAG1-induced degradation of NOTCH3 WT but not that of R141C and C185R.***A*, images of immunocytochemistry with an N3ECD antibody after coculturing N3WT/C185R-RF-HeLa with MIG-3T3 or JAG1-3T3 cells. N3WT/C185R-RF-HeLa was cultured with and without dox for 1 day before being cocultured with MIG-3T3 or JAG1-3T3 for 1 day. The *left panel* shows GFP expressed in MIG-3T3 and JAG1-3T3 (*green*). The *middle panel* shows N3ECD (*magenta*). The *right panel* shows an overlay. *Arrows* indicate N3ECD transendocytosed into JAG1-3T3. The scale bar represents 10μm. *B*, proportion of JAG1-3T3 and MIG-3T3 positive with NOTCH3 WT and C185R (independent biological replicates, N = 4). *C*, representative images of immunocytochemistry with an N3ECD antibody after coculturing N3WT/R141C-RF-HeLa with JAG1-3T3 cells. N3WT/R141C-RF-HeLa was cultured with and without dox for 1 day before being cocultured with MIG-3T3 or JAG1-3T3 for 1 day. The scale bar represents 10μm. *D*, proportion of JAG1-3T3 and MIG-3T3 positive with NOTCH3 WT and R141C (independent biological replicates, N = 5). Data information: In *B* and *D*, data are presented as mean ± SD. ∗*p* < 0.05, ∗∗*p* < 0.01, ∗∗∗*p* < 0.001, ∗∗∗∗*p* < 0.0001, n.s., not significant (Tukey’s *post hoc* test following three-way ANOVA). JAG1, Jagged 1; N3ECD, NOTCH3 extracellular domain; RFNG, Radical fringe.

**Figure 7:**
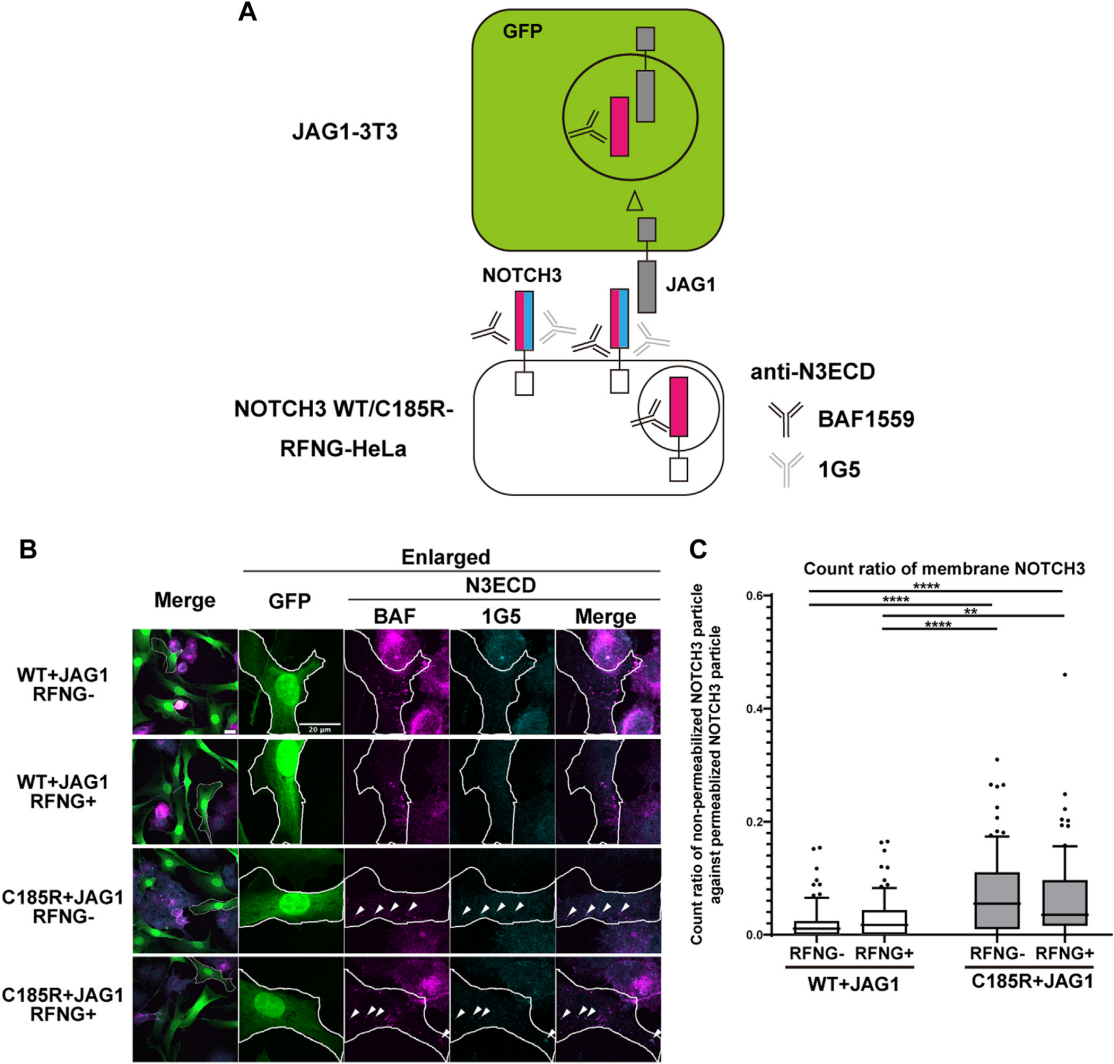
**RFNG does not affect JAG1-mediated transendocytosis of NOTCH3 WT and C185R.***A*, schematic representation of the immunocytochemistry workflow used to separately detect NOTCH3 present in the cell membrane and in JAG1-3T3 cells. After N3WT/C185R-RF-HeLa cells transfected with NOTCH2 siRNA were cocultured with JAG1-3T3 cells, the fixed cells were stained with two different anti-NOTCH3ECD (N3ECD) antibodies (clones 1G5 and BAF1559) before and after cell permeabilization, respectively. The scale bars indicate 20 μm. *B*, representative confocal images of cocultured N3WT/C185R-RF-HeLa and JAG1-3T3 cells, as depicted in (A). The *white arrowheads* indicate NOTCH3 particles detected by antibodies against N3ECD (clones 1G5 and BAF1559), indicating the presence of NOTCH3 on the cell membrane. The scale bars indicate 20 μm. *C*, the ratio of NOTCH3 present on the cell membrane against the total NOTCH3 in JAG1-3T3 cells. Box plots: 10 to 90 percentile. (independent biological replicates: N = 3; total cell numbers, *WT RFNG-: n = 86, WT RFNG+: n = 86, C185R RFNG-: n = 93, C185R RFNG+: n = 81). ∗∗p < 0.01,* ∗∗∗∗*p* < 0.0001 (Tukey’s *post hoc* tests following two-way ANOVA). JAG1, Jagged 1; N3ECD, NOTCH3 extracellular domain; RFNG, Radical fringe.

To examine whether RFNG affects ligand-dependent endocytosis and degradation of NOTCH3 WT and C185R, we conducted a coculture experiment using N3WT/C185R-RF-HeLa and JAG1-3T3 cells. After 24 h, we performed the immunocytochemistry with an anti-NOTCH3ECD antibody. NOTCH3 WT and C185R were observed in JAG1-3T3 cells but not in parental control cells (Fig. 6*A*). For quantification, we calculated the percentage of N3ECD-positive JAG1-3T3 or MIG-3T3 (Fig. 6*B*). RFNG induction reduced the percentage of NOTCH3 WT-positive JAG1-3T3 cells from ∼35% to ∼15% (Fig. 6*B*, left) but had no effect on NOTCH3 C185R-positive cells (Fig. 6*B*, right). By contrast, RFNG did not significantly affect the endocytic pathway of NOTCH3 WT and C185R upon binding to DLL4 (Fig. S5, *A* and *B*). To assess JAG1-dependent degradation of NOTCH3 R141C, a mutant in EGFr3, we generated HeLa cell lines expressing NOTCH3 WT or R141C at similar activity levels (Fig. S8*A*). Similar to C185R, NOTCH3 R141C remained unaffected by RFNG (Fig. 6, *C* and *D*). We further investigated whether RFNG inhibits transendocytosis or promotes degradation of NOTCH3 WT after endocytosis by JAG1. To address this, we cocultured cells with leupeptin, a lysosomal protease inhibitor, to block the lysosomal degradation of NOTCH3 after JAG1-mediated endocytosis (17). Furthermore, considering that clustering of NOTCH1 is observed before its transendocytosis (66), NOTCH3 interacts heterodimerically with other NOTCH receptors (67, 68), and HeLa cells endogenously express NOTCH2 (69), we explored the potential impact of NOTCH2 on JAG1-dependent transendocytosis of NOTCH3. First, we confirmed *NOTCH2* knockdown efficiency by dual luciferase reporter assays, and it showed that JAG1-mediated NOTCH activity was completely abrogated by NOTCH2 knockdown in parental HeLa cells (Fig. S6, *A* and *B*). Subsequently, we conducted immunocytochemistry with anti-N3ECD antibodies after coculturing N3WT/C185R-RF-HeLa and JAG1-3T3 cells for 8 h with leupeptin treatment, following *NOTCH2* gene knockdown. In the immunostaining, we used two anti-N3ECD antibodies before and after cell membrane permeabilization to detect NOTCH3 remaining at the cell membrane and those taken into the cell separately (Fig. 7, *A* and *B*). Quantification revealed a significantly elevated ratio of membrane-bound NOTCH3 C185R compared to NOTCH3 WT, irrespective of RFNG expression (Fig. 7*C*). This suggests impaired JAG1-mediated transendocytosis of NOTCH3 C185R, consistent with a previous report (35). However, RFNG did not alter the membrane ratio of NOTCH3 WT and C185R (Fig. 7*C*), indicating that RFNG did not reduce JAG1-mediated transendocytosis of NOTCH3 WT and C185R. Taken together, despite the minimal impact of RFNG on NOTCH3 WT and C185R transendocytosis efficacy, RFNG facilitated JAG1-dependent degradation of NOTCH3 WT following endocytosis. However, this effect was not exerted on R141C and C185R mutants. These findings suggest that as compared to NOTCH3 WT, RFNG confers resistance of NOTCH3 R141C and C185R to degradation within the JAG1-expressing cells, rather than to endocytosis.

### RFNG represses JAG1-mediated signaling activity of NOTCH3 R141C and C185R more effectively than WT

**Figure 8:**
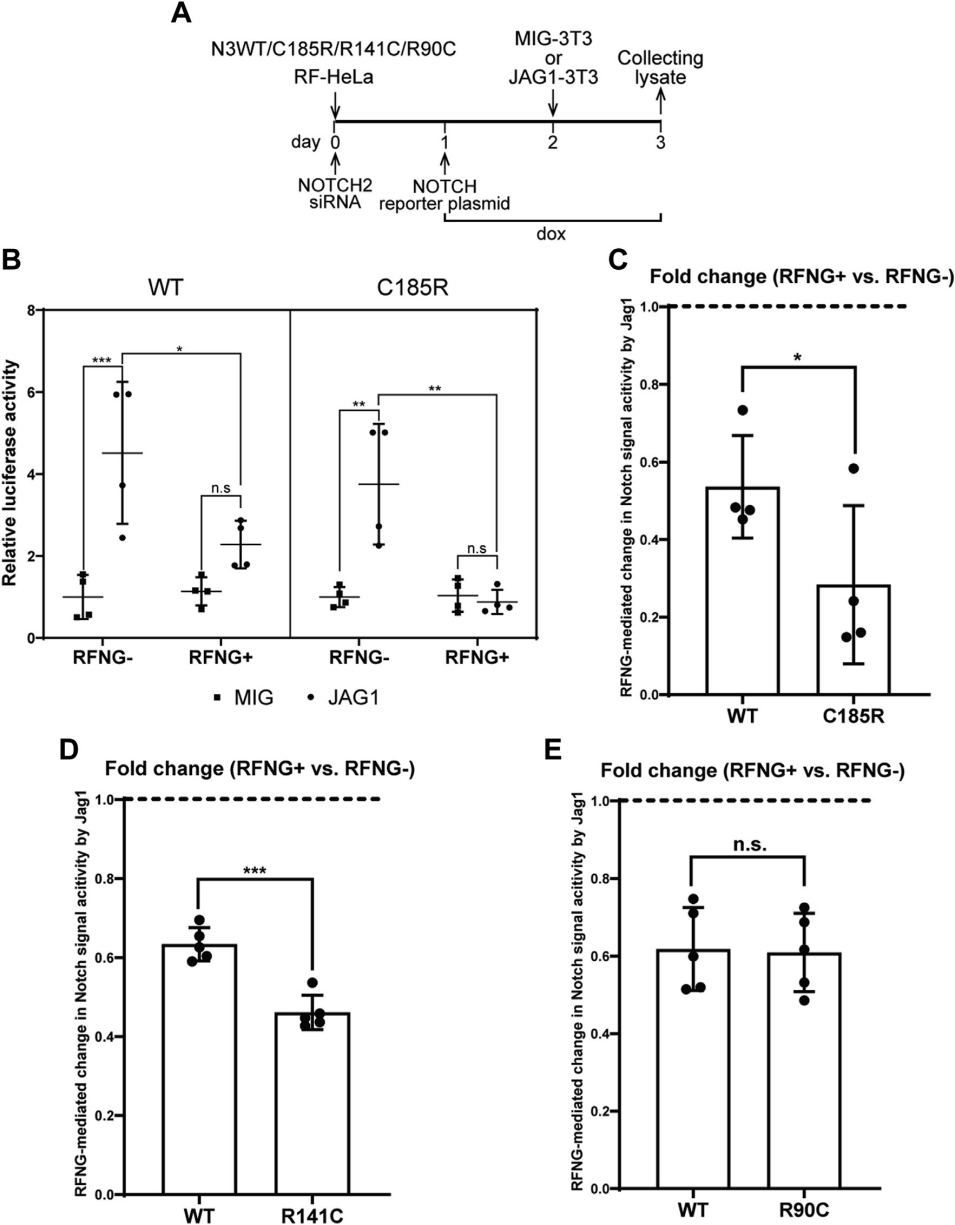
**RFNG impairs JAG1-stimulated signaling activity of NOTCH3 R141C and C185R more effectively than WT.***A*, a workflow for evaluating the JAG1-mediated signaling activity of NOTCH3 WT and C185R, with NOTCH2 knockdown. N3WT/C185R-RF-HeLa cells were transfected with NOTCH2 siRNA on day 0. The cells were transfected with the Notch reporter plasmid, and doxycycline was added or not added on day 1. Subsequently, MIG-3T3 or JAG1-3T3 cells were seeded on day 2, and total cell lysates were collected on day 3. *B*, Notch reporter assay with the collected lysates from cocultures of N3WT/C185R-RF-HeLa and JAG1-3T3 or MIG-3T3 cells. Values were calculated relative to that observed upon coculture with MIG-3T3 cells (independent biological replicates N = 4). *C*, fold change in JAG1-mediated activities of NOTCH3 WT and C185R with RFNG coexpression against those without RFNG (independent biological replicates N = 4). *D*, fold change in JAG1-mediated activities of NOTCH3 WT and R141C with RFNG coexpression against those without RFNG (independent biological replicates N = 5). *E*, fold change in JAG1-mediated activities of NOTCH3 WT and R90C with RFNG coexpression against those without RFNG (independent biological replicates N = 5). Data information: In *B*-*G*, data are presented as mean ± SD. n.s., not significant, ∗*p* < 0.05, ∗∗*p* < 0.01, ∗∗∗*p* < 0.001, ∗∗∗∗*p* < 0.0001 (B, D, and F: Tukey’s *post hoc* test following three-way ANOVA; C, E, and G: Unpaired one-tailed Student’s *t* test). JAG1, Jagged 1; RFNG, Radical fringe.

Additionally, NOTCH3 signaling activity plays a critical role in mural cell identity and cerebral vascular homeostasis (29, 30, 31, 32, 34). Fringe modulates NOTCH1 and NOTCH2 signaling activity in response to JAG and DLL (37, 38, 39, 40, 41). To investigate whether RFNG regulates the JAG1-mediated signaling activity of NOTCH3 WT and C185R, we performed a coculture system of N3WT/C185R-RF-HeLa cells with JAG1-3T3 or MIG-3T3 cells with siRNA-mediated NOTCH2 knockdown (Fig. 8*A*). Subsequently, we analyzed a Notch reporter assay using whole lysates from the cocultured cells (Fig. 8*B*). Compared to parental cells, JAG1 induced NOTCH3 WT signaling, which RFNG reduced by approximately half (Fig. 8*B*). In contrast, RFNG almost completely inhibited NOTCH3 C185R activity (Fig. 8*B*). In other words, RFNG-mediated inhibition of the JAG1-stimulated activity of NOTCH3 C185R was pronounced than that observed for WT (Fig. 8*C*). We also evaluated *HES1* expression, a NOTCH target gene, in the coculture system. JAG1-stimulated activation of NOTCH3 C185R upregulated *HES1* expression, which RFNG significantly repressed (Fig. S7). Furthermore, we examined the effect of RFNG on JAG1-mediated signaling in NOTCH3 other mutants: R90C, which is a mutant in EGFr2, and R141C. We established HeLa cell lines expressing these mutants at comparable activity levels with WT (Fig. S8, *A* and *B*). RFNG exerted a higher inhibitory effect on the activation of NOTCH3 R141C than that of WT (Figs. 8*D* and S8*A*), while its impact on R90C was negligible (Figs. 8*E* and S8*B*). On the other hand, RFNG enhanced the DLL4-dependent signaling activity of both NOTCH3 WT and C185R (Figs. S6*C* and S9*A*), with no significant difference between them (Fig. S9*B*). Collectively, RFNG selectively impaired JAG1-dependent signaling transduction through NOTCH3 R141C and C185R, with distinct effects on NOTCH3 WT and R90C.

## Discussion

In this study, we investigated the impact of RFNG on the glycosylation, JAG1-and DLL4-dependent degradation, and signaling activation of three NOTCH3 CADASIL mutants, R90C, R141C, and C185R, corresponding to EGFr2, 3, and 4, respectively. We found that RFNG is involved in defects in JAG1-dependent degradation and signaling activation of NOTCH3 R141C and C185R compared to NOTCH3 WT.

**Figure 9:**
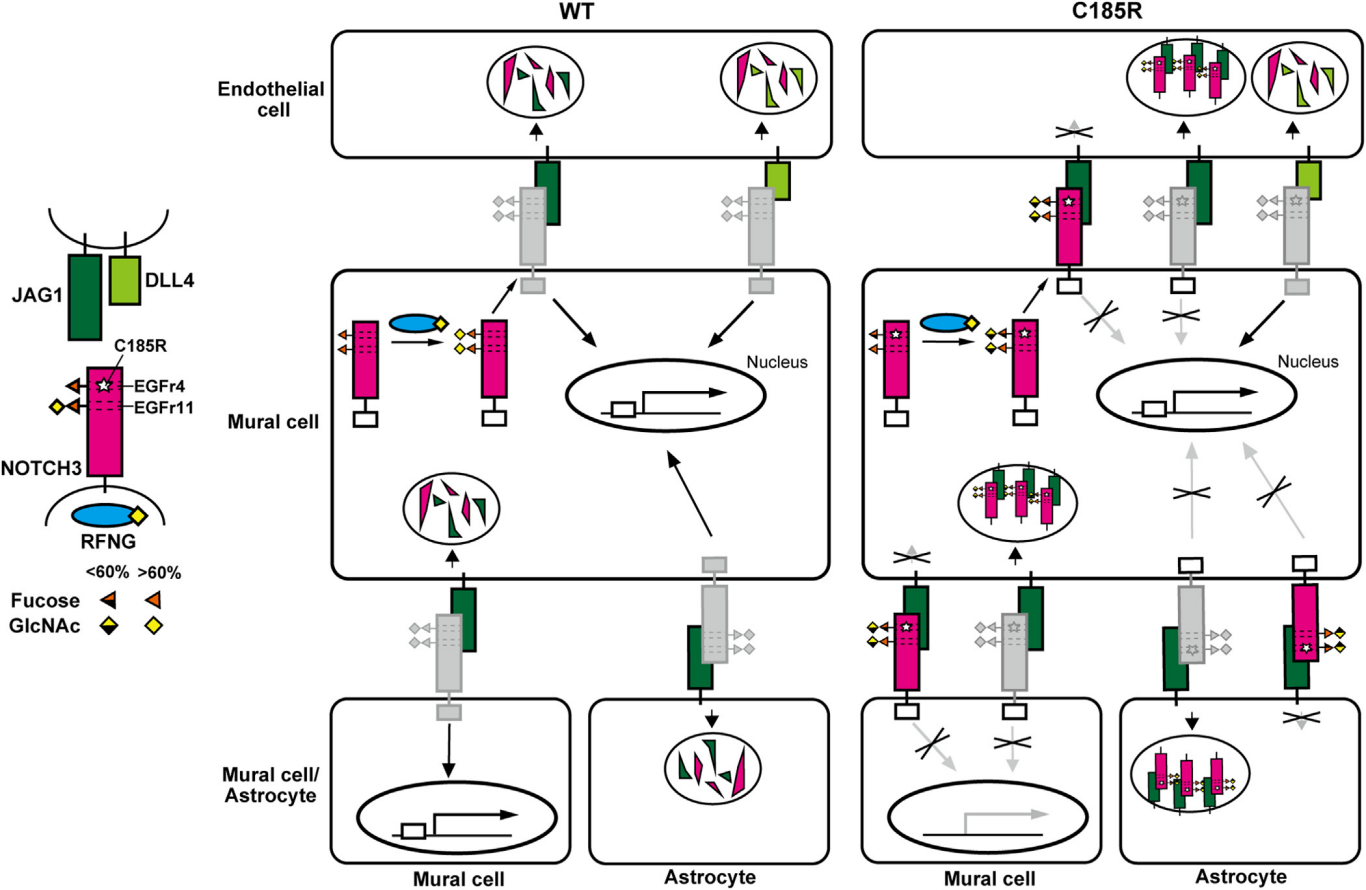
**Illustration of a hypothetical model for the signal impairment and accumulation of the NOTCH3 C185R mutant protein**.

Based on the available expression data for *Notch3*, *Rfng*, *Jag1*, and *Dll4* in mouse endothelial cells, mural cells, and astrocytes (Fig. S1), our study significantly advances our understanding of the vascular cell communications involved in the accumulation and signaling impairment of NOTCH3 C185R mutant proteins compared to NOTCH3 WT (Fig. 9). NOTCH3 C185R expressed in mural cells, alongside coexpressed RFNG, may be activated by DLL4 expressed in endothelial cells to a similar extent as NOTCH3 WT but may exhibit reduction in JAG1-regulated activity (Fig. 9). On the other hand, mural cells in the cerebral vasculature contact each other (70, 71), and astrocytic endfeet associate with the microvasculature (72). Both mural cells and astrocytes express JAG1 but not DLL4 (Fig. S1, *H* and *I*) (49). Consequently, upon interaction with JAG1, NOTCH3 C185R may exhibit prolonged stability within endothelial cells, mural cells, and astrocytes. Simultaneously, the NOTCH3 mutant protein may represent reduced activity in mural cells (Fig. 9). By contrast, proteomic analysis of brain lysates from *postmortem* patients and animal models indicated that JAG1 is not included in the GOMs (73). JAG1 may be involved in the accumulation of NOTCH3 C185R but not in the further formation of GOMs. Additional research is necessary to determine the vascular cell-cell interaction during GOM aggregation.

Our LC-MS/MS analysis revealed that RFNG affects the *O-*fucose glycan state of NOTCH3 WT and C185R (Fig. 2*D*). The NOTCH3 C185R fragment exhibited structural changes and greater multimerization than did the WT under nonreducing conditions, which is consistent with previous reports (22, 23, 24, 26). This abnormal state may lead to reduced glycosylation of the *O-*fucose consensus site in EGFr4 (Fig. 2*D*), mediated by POFUT1 (37, 38). Furthermore, compared with the WT, the C185R mutation partially inhibited the RFNG-mediated modification of *O-*fucose in EGFr4 but did not affect *O-*fucose in EGFr11. This finding suggested that the CADASIL mutation could reduce the elongation rate of *O-*fucose in an EGFr with its mutation but not in the other remote domains. However, *O-*fucose glycans in EGFr other than EGFr4 and 11 remain unexplored due to technical limitations. In future work, whether RFNG affects the other *O-*fucose sites of NOTCH3 mutants is to be examined using digestive enzymes other than trypsin.

While NOTCH1 extracellular domain transendocytosis is crucial to NOTCH1 signaling activation (15), RFNG did not significantly impair transendocytosis of NOTCH3 WT and C185R in our experimental condition. We cannot exclude the possibility that RFNG induces only a modest change in NOTCH3 transendocytosis, which our experimental condition could not detect. Alternatively, Bian *et al.* have reported that insufficient supplementation of NOTCH1 from ERGIC, endoplasmic reticulum-Golgi intermediate compartment, to the cell membrane causes defects in NOTCH1 activation (74). The question arises whether RFNG’s impact on NOTCH3 R141C and C185R involves abnormal accumulation in endoplasmic reticulum-Golgi intermediate compartment rather than the cell membrane, affecting signaling transduction. By contrast, Handa *et al.* have demonstrated that NOTCH1 signaling activation stimulated by immobilized DLL4 magnetic beads, which do not induce transendocytosis, involves actin cytoskeleton (75). These possibilities should be explored in future research.

NOTCH3 signaling plays a crucial role in the differentiation and survival of VSMCs and pericytes (28, 29, 30, 31). Previous studies have suggested that JAG1-NOTCH3 signaling contributes to the proper development and function of vascular mural cells in mouse embryos and retinas (43, 44, 45). Recently, Romay *et al.* have reported that JAG1-NOTCH3 signaling declines in aged mouse mural cells, and NOTCH3 null mutation leads to chronic hypoperfusion, proteoglycan accumulation in brain parenchyma, and upregulation of neurodegenerative markers (32). Our findings suggest that RFNG-mediated reduction in NOTCH3 R141C and C185R activities, compared to WT, could impair mural cell development, cerebrovascular function, and protein clearance in CADASIL patients with these mutations. In previous research, NOTCH3 R133C, a mutant in EGFr3, exhibits less activation by JAG1 in VSMCs from CADASIL patients than in healthy control cells (33). However, in rat aortic smooth muscle cells, NOTCH3 R133C activation is similar to NOTCH3 WT (36). This discrepancy might be attributed to varying RFNG expression levels across distinct cell types. Additionally, RFNG inhibits NOTCH3 R90C signaling to a similar degree as WT. In accordance with this, overexpression of NOTCH3 WT and R90C rescues NOTCH3 KO-induced abnormalities in the vasculature *in vivo* (76). Therefore, even if the RFNG-mediated mechanism could not explain the functional changes of all NOTCH3 CADASIL mutant proteins, it can be argued that RFNG is involved in decreased activity of NOTCH3, at least in part, EGFr3 and 4 mutants. These results may account for a larger population size of carriers with mutations in EGFr3 and 4 than those in the other domain (21). Further studies are required to explore the effects of RFNG on other NOTCH3 mutants.

Our coculture experiment also revealed that RFNG promotes JAG1-dependent degradation of NOTCH3 WT but not in NOTCH3 R141C and C185R. This indicates that the NOTCH3 mutants in EGFr3 and 4 are relatively susceptible to accumulation in JAG1-expressing cells under conditions of high RFNG expression. Furthermore, sucrose gradient experiments revealed that RFNG altered the density of NOTCH3 C185R fragments but not WT. Hiruma-Shimizu *et al.* suggested that the Fringe-mediated elongation of *O-*fucose with GlcNAc in the NOTCH1 EGFr12 leads to conformational changes (77). Nevertheless, the preclustered soluble NOTCH3 EGFr1-12 WT and C185R protein was significantly degraded by JAG1 regardless of RFNG expression. This discrepancy may be explained by the artificial clustering of NOTCH3 fragments and the difference between NOTCH3 full-length and EGFr1-12 fragments. Future studies should clarify the mechanism of RFNG-mediated resistance to protein degradation in NOTCH3 R141C and C185R.

Although Viitanen *et al.*found similar mRNA levels of cyclin D, a senescence marker, in cultured VSMC cells from CADASIL mutation R133C carriers and healthy controls (78), aging remains crucial in GOM accumulation and CADASIL progression (8, 11, 12). The age-related aggregation of NOTCH3 mutant protein may be explained by our study using immortalized pericyte cell lines, which demonstrates that senescence induced by long-term culture increased transcript levels of NOTCH3 and RFNG. Although confirmation is needed regarding NOTCH3 protein levels and RFNG-mediated glycosylation of NOTCH3 with age *in vivo*, targeting RFNG-mediated elongation of *O-*fucose in NOTCH3 mutant proteins could be a therapeutic strategy to prevent GOM formation and mitigate CADASIL pathogenesis in patients with some mutations in NOTCH3 EGFr3 and 4.

## Experimental procedures

### Constructs and transfection

The RFNG plasmid was produced by amplifying the RFNG short fragment (ENST00000429557.7, RFNG-202) from the HBPC/ci37 complementary DNA and RFNG202 PCR primers using Blend-Taq Plus (TOYOBO). The amplified fragment was inserted into pENTR1A (pENTR1A-RFNG202). pCLI-hRFNG208 (RIKEN Bio Resource Center, DB462374; ENST00000582478.5) was purchased from RIKEN BioResource Center. RFNG-208 and RFNG-202 have overlapping regions. To obtain the RFNG full-length fragment (ENST00000310496.9, RFNG201), overlap PCR was performed with KOD -Plus- Neo (TOYOBO) using pENTRA1A-RFNG202 and pCLI-hRFNG208 as templates. The obtained fragment was inserted into pENTRA1A, but the resulting sequence did not include a portion of the desired sequence (pENTR1A-RFNG201D-HA). To fill in the missing sequence, inverse PCR was performed with KOD -Plus- Neo (TOYOBO) using pENTR1A-RFNG201D-HA as a template, after which pENTR1A-RFNG201 was produced. The RFNG201-HA fragment was cloned into pINDUCER20 by Gateway Technology (Invitrogen) to produce pINDUCER20-RFNG-HA (61). NOTCH3 EGFr1-12 WT/C185R expression plasmids were prepared by cloning NOTCH3 EGFr1-12 WT and C185R amplified by PCR from pCMV-hNOTCH3 WT (67) and C185R (27) into pCAGGS-S3AsM-FcHis (79) by Seamless Ligation Cloning Extract, as described in a previous study (80). The PCR primers used for these constructions are listed in Table S1. These constructs were transfected into cells with PEI 25K or PEI MAX (Polysciences), according to the manufacturer’s instructions.

### Lentivirus

HEK293T cells were cotransfected with pINDUCER20-RFNG201, pCAG-HIVgp, and pCMV-VSV-G-RSV-Rev. The supernatant containing the RFNG-expressing lentivirus was collected after centrifuging the medium 2 days posttransfection. The RFNG-expressing lentivirus was transduced into cells with 10 mg/ml polybrene (Nacalai Tesque).

### siRNA

siRNAs for human NOTCH2 (sense: 5′-GGAGGUCUCAGUGGAUAUATT-3′, antisense: 5′-UAUAUCCACUGAGACCUCCTT-3′) were synthesized by Japan Bio Service and transfected into cells with Lipofectamine RNAiMAX Transfection Reagent according to the manufacturer’s instructions.

### Cell lines

HBPC/ci37 cells were cultured in Pericyte medium supplemented with 2% fetal bovine serum (FBS) and 1% penicillin/streptomycin solution (ScienCell) at 33 °C and 5% CO2 to proliferate or at 37 °C to allow differentiation (55). A stable HEK293 cell line in which RFNG expression was regulated by doxycycline (HEK293-RFNG) was established by transducing the RFNG-expressing lentivirus into HEK293 cells and cloning it with 500 μg/ml G418 hydrochloride (Nacalai Tesque). Stable HeLa cell lines expressing NOTCH3 WT/C185R and RFNG (N3WT/R90C/R141C/C185R-RF-HeLa) were generated by transducing the RFNG-expressing lentivirus into HeLa cell lines that constitutively expressed NOTCH3 WT, R90C, R141C, or C185R according to the initiation region/matrix attachment region method (81), as established in our previous study (27), and selecting them with 500 μg/ml G418. The NIH-3T3 cell lines expressing JAG1 and DLL4 and the parental controls (JAG1-3T3, DLL4-3T3, and MIG-3T3) were gifts from Dr Hozumi (Tokay University) (65). These HEK293, HeLa, and NIH-3T3 cell lines were cultured in Dulbecco's modified Eagle's medium (DMEM) (high glucose) supplemented with L-glutamine and phenol red (Nacalai Tesque), 10% FBS (Gibco, SRN, Nichirei) and 1% penicillin streptomycin (Nacalai Tesque) at 37 °C and 5% CO2.

### Senescence-associated β-galactosidase staining

After HBPC/ci37 cells (4.3 × 10∧4/35-mm dish) were cultured for 1, 7, or 14 days at 37 °C in serum-depleted medium, they were fixed with 4% paraformaldehyde (PFA) in PBS. The cells were stained with X-gal solution (10% X-gal, 5 mM potassium ferrocyanide, 5 mM potassium ferricyanide, 150mM NaCl, 2 mM MgCl2, and 40 mM citrate-phosphate buffer at pH 6.0) for 4 h at 37 °C. Thereafter, we washed the cells with diluted water and captured images with an optical microscope (ECLIPSE Ts2, Nikon). The number of stained cells was counted by Fiji (NIH) with the plugin Cell Counter and calculated relative to the total number of cells.

### Quantitative PCR

For Figures 1 and S1, HBPC/ci37 cells were cultured in serum-depleted medium for 1, 7, or 14 days at a density of 1.0 × 10^5^/60-mm dish. HeLa cells were transfected with NOTCH2 siRNA and cultured for 16∼24 h at ∼70% confluency in a 35-mm dish. For Fig. S7, N3C185R-RF-HeLa (0.7 × 10∧4) was precultured with and without 1μg/ml doxycycline for about a day, following a coculture with MIG-3T3 or JAG1-3T3 (2.0 × 10∧5) for 24 h. Total RNA was extracted from the cells using Sepasol-RNA I Super G (Nacalai Tesque) according to the manufacturer's instructions. Complementary DNA synthesis was performed using ReverTra Ace (TOYOBO). The mRNA expression levels of each gene were quantified *via* real-time PCR with THUNDERBIRD SYBR qPCR Mix (TOYOBO) on a LightCycler 96 System (Roche). The results are shown as the ΔCt or ΔΔCt method using GAPDH, PUM1, and GUSB as reference genes. The sequences of primers used are listed in Table S2.

### Purification of NOTCH3 EGFr1-12 fragments

HEK293-RFNG cells were cultured in 100-mm dishes until they reached ∼70% confluency. The cells were treated with 1 μg/ml doxycycline or left untreated. One day after transfection with pCAGGS-NOTCH3 EGFr1-12 WT or C185R-Fc-His, the cells were exchanged with fresh serum-depleted DMEM. To purify the NOTCH3 EGFr1-12 WT/C185R-Fc-His, the medium was centrifuged to remove cell debris after 6 days post transfection, after which the mixture was incubated with Ni Sepharose 6 Fast Flow Mix (GE HealthCare) overnight. The beads were washed with phosphate buffer containing 40 mM imidazole and eluted with phosphate buffer containing 500 mM imidazole to obtain purified NOTCH3 EGFr1-12 proteins. The concentrations of the purified proteins were determined by SDS‒PAGE and gel staining with CBB Stain One Super (Nacalai Tesque).

### Liquid chromatography with tandem mass spectrometry

For in-solution digestion, purified NOTCH3 EGFr1-12 was subjected to a reduction by incubating the purified protein in 8 M urea/10 mM Tris(2-carboxyethl)phosphine hydrochloride/400 mM NH_4_HCO_3_ at 50 °C for 5 minutes. Following reduction, the samples were alkylated with 350 mM iodoacetamide/50 mM Tris–HCl for 30 min in the dark. Reduced and alkylated proteins were digested with 5 ng/ml trypsin gold in 20 mM diammonium phosphate for 4 h at 37 °C. The solution containing the digested peptides was desalted with ZipTip (Millipore) and dissolved in 10% acetonitrile in 0.1% trifluoroacetic acid.

The peptides were analyzed using an Orbitrap Fusion Tribrid mass spectrometer (MS) (Thermo Fisher Scientific) coupled to an UltiMate 3000 RSLC nano-LC system (Dionex Co with Technos) *via* a nanoelectrospray ionization source. The LC gradient of a nano-HPLC capillary column (150 mm × 75 μm, Nikkyo) with 0.1% formic acid as solution A and 0.1% formic acid 90% acetonitrile as solution B was set as follows: 5%–100% B (0–45 min), 100%–5% B (45–45.1 min), and 5% B (45.1–60 min). Data-dependent tandem MS analysis was performed using a top-speed approach (cycle time of 3 s). Precursor ions were analyzed with an Orbitrap mass analyzer, whereas fragment ions generated by higher-energy collisional dissociation fragmentation were analyzed with an Orbitrap or a linear ion trap mass analyzer.

### Quantitative and semiquantitative analysis of O-fucose glycan

Peak lists were generated using the extract MSN in Xcalibur 3.0.6 (Thermo Fisher Scientific, https://www.thermofisher.com/order/catalog/product/OPTON-30965) with the default parameters. The data analysis was performed using Byonic software (version 4.5.2, https://proteinmetrics.com/byonic/). To generate an *in silico* glycopeptide digest library, the following input files and parameters were used: human NOTCH3 [UniProt ID: Q9UM47] amino acid sequence 1 to 505 as peptide sequences; Trypsin (specific for cleavage at the C terminus of Lys/Arg) as a proteolytic enzyme with the possibility of zero missed cleavage; carbamidomethylation on Cys residues as a fixed modification; and oxidation on Met, Asp, or Asn residues as a variable modification. The following glycan lists were also specified as variable modifications: Hex(1) at the POGLUT2/3-dependent O-Glc site on EGFr4; Fuc(1), HexNAc(1)Fuc(1), HexNAc(1)Hex(1)Fuc(1), and HexNAc(1)Hex(1)Fuc(1)NeuAc(1) at *O*-Fuc sites on EGFr4 and 11; and MS/MS analysis was performed with the following parameters: MS1 tolerance, 20 ppm; MS/MS tolerance, 0.5 Da; and quadrupole time-of-flight/higher-energy collisional dissociation mode. For the inspection of MS/MS spectra, correct assignments of major b/y ions and characteristic glycan-derived fragment ions at 138, 168, 186, 204, 274, 292, 366, 512, and 657 m/z were manually inspected using the Byonic and Xcalibur Qual browsers (version 4.2, Thermo Fisher Scientific).

If particular glycoforms were detected in only 1 of the 2 replicates, the other replicate was subjected to manual data analysis based on the expected retention time to check for reproducibility. The MS/MS spectra of EGFr4 and 11 are shown in Tables S3 and S4, respectively.

The extracted ion chromatograms were generated using the Xcalibur Qual browser by selecting the most abundant isotopic peaks (monoisotopic or second isotope peaks) with Gaussian smoothing at 5 points. The extracted ion chromatogram peak height was measured to compensate for the difference in isotope abundance. The relative intensity derived from all the observed charge states of each glycopeptide was summed. The proportion of integrated peak height values for specified glycoforms to those corresponding to all detectable (glyco-) peptides was calculated for semiquantification.

### Western blotting

The protein and lysate samples were separated by SDS‒PAGE and transferred to polyvinylidene fluoride membranes (Merck Millipore). After the membranes were stained with ponceau solution (0.001% (w/v) ponceau S and 1% acetic acid) for approximately 15 min at room temperature (RT), we examined the total protein concentration by scanning the membrane. The membranes were then washed with Tris buffered saline (TBS) with 0.1% Tween-20, blocked with 0.3% skim milk in TBS for an hour at RT, and incubated overnight at 4 °C with 0.3% skim milk in TBST containing the following primary antibodies: mouse anti-human IgG Fcγ (Jackson ImmunoResearch, 1:1000), mouse anti-human NOTCH3ECD (Abnova, 1G5; 1:1000), mouse anti-human/mouse α-TUBULIN (Wako, 10G10; 1:4000), mouse anti-HA (Covance, 16B12; 1:1000), mouse anti-RFNG (SantaCruz, 18-K2; 1:1000), and mouse anti-FLAG M2 (Sigma-Aldrich; 1:1000). After three washes with TBST, the membrane was treated with peroxidase conjugated AffiniPure goat anti-mouse IgG (Jackson ImmunoResearch; 1:20,000) in TBST for an hour at RT. Thereafter, the membrane was washed with TBST three times and then treated with enhanced chemiluminescence solution. Chemiluminescence was detected with an Ez-Capture MG system (ATTO). Band intensities were quantified by a CS analyzer 3.0 (ATTO). Total protein was quantified by staining with ponceau in Fiji (NIH) with the Band Peak Quantification plugin.

### Nonreducing SDS‒PAGE

The purified NOTCH3 EGFr1-12 fragments were boiled in 2 × Laemmli sample buffer supplemented with or without 2-mercaptoethanol and subjected to Western blotting as described above. NOTCH3 band intensities corresponding to dimers and multimers were calculated relative to the total intensities from nonreducing SDS‒PAGE results.

### Sucrose gradient ultracentrifugation assay of NOTCH3 fragments

We adjusted the concentration of purified NOTCH3-EGFr1-12 to 6 ng/μl with PBS and added 100 μl of the mixture to a sucrose density gradient prepared by loading 200 μl of 50%, 40%, 30%, 20%, and 10% sucrose solution in PBS from the bottom to the top of a 3-ml PC tube (Hitachi, Koki) (82). Preparations were ultracentrifuged at 50,000 rpm at 4 °C for 4 h in a swing rotor P55ST2 (Hitachi, Koki) in a CP 80NX (Hitachi, Koki). Then, we collected 12 fractions by carefully taking 100μl from the top to the bottom (fractions 1–11) and mixing the pellet with 100 μl of PBS (fraction 12). All the fractions were mixed with 5 × Laemmli sample buffer and boiled at 95 °C for 5 min. These samples were subjected to Western blotting as described above using mouse anti-human NOTCH3ECD (Abnova, 1G5, 1:1000) as a primary antibody. We calculated the percentages of NOTCH3 band intensities in each fraction against the total amount in all the fractions by a CS-analyzer (ATTO). The ratio of the relative NOTCH3 amount in fractions 7 and 8 to that in fraction 5 was subsequently calculated. Three independent experiments were conducted using three lots of purified NOTCH3 fragments.

### Binding and uptake of the purified NOTCH3 EGFr1-12 fragment

MIG-3T3 and JAG1-3T3 cells were seeded on cover slips coated with collagen type I-C (Nitta Gelatin) at a density of 4 × 10^5^ cells/well in a 6-well plate and cultured overnight. The concentrations of the purified NOTCH3 EGFr1-12 proteins were adjusted to 4 nM with anti-hFc (Biotin-SP AffiniPure Goat Anti-Human IgG, Fc (gamma) Fragment, Jackson ImmunoResearch) and 2% FBS (Nichirei, SRN) in DMEM, after which the proteins were clustered at 37 °C for 30 min. Preclustered NOTCH3 was incubated with the cells on coverslips on ice for 40 min, after which the cells were washed with cold Hanks' balanced salt solution. The cells were lysed in radioimmunoprecipitation assay buffer to evaluate binding. For the endocytosis of the NOTCH3 fragments, the cells were incubated in DMEM supplemented with 2% FBS at 37 °C and 30 min and lysed. The collected lysates were boiled with 5 × Laemmli sample buffer at 95 °C for 5 min and subjected to Western blotting as described above. NOTCH3 band intensities were normalized to the total protein staining. At least two lots of purified NOTCH3 proteins were used in three independent experiments.

### Puromycin chase assay

N3WT/C185R-RF-HeLa cells were precultured in a 35-mm dish at a density of 2 × 10^5^ cells/dish with or without 1 μg/ml doxycycline added to the medium. Thereafter, the cells were incubated with 150 μg/ml puromycin at 37 °C and CO_2_ for 0 or 16 h and lysed in Triton lysis buffer (50 mM Tris–HCl (pH 7.5), 150 mM NaCl, 1% Triton X-100, 12.5 mM b-glycerophosphate, 10 mM NaF, 2 mM DTT, 1% protease inhibitor cocktail, and 10 mM NEM). The collected lysates were boiled with 2 × Laemmli sample buffer at 96 °C for 5 min and subjected to Western blotting as described above. The band intensities of the N3ECD at 16 h post treatment with puromycin were calculated relative to those at 0 h.

### Luciferase reporter assay

To evaluate the JAG1-stimulated signaling activity of NOTCH3, N3WT/R90C/R141C/C185R-RF-HeLa cells were transfected with 10 pmol of NOTCH2 siRNA and seeded into a 12-well plate at a density of 5.0 × 10^4^ cells/well. The cells were then cultured overnight. The Notch reporter plasmid (pGL4.1-TP1-Emerald luciferase (Eluc), 0.8 μg/well) and Renilla luciferase (Rluc) plasmid (pRL-TK, 0.0016 μg/well) were transfected into the cells, which were then replaced with fresh medium supplemented with or without 1 μg/ml doxycycline after 4 h post transfection. N3WT/R90C/R141C/C185R-RF-HeLa cells were cocultured with MIG-3T3 or JAG1-3T3 cells at a density of 8 × 10^4^ cells/well for 24 h and then lysed in passive lysis buffer (Promega). Eluc and Rluc activities were determined with the Dual-Luciferase Reporter Assay System (Promega) according to the manufacturer’s instructions. Eluc activity was divided by Rluc activity for normalization of the transfection efficiency. Therefore, Notch luciferase reporter activity was calculated relative to that observed after coculture with MIG-3T3 cells and without doxycycline. Inhibitory effect of RFNG on Notch activity was calculated as a fold change of Notch activity with RFNG relative to that without RFNG and minus one.

To examine DLL4-induced signaling activity, N3WT/C185R-RF-HeLa cells were cultured in a 12-well plate at a density of 8.0 × 10^4^ cells/well with or without 1 μg/ml doxycycline for 1 day and cocultured with MIG-3T3 or DLL4-3T3 cells at a density of 8.0 × 10^4^ cells/well for an additional day. Then, the cell lysates were collected and subjected to a dual-reporter assay. Enhancement effect by RFNG on Notch activity was calculated as fold change of relative Notch activity with RFNG relative that without RFNG and minus one.

### Immunocytochemistry

As for Figure 6 and Fig. S5, HeLa-N3WT/R141C/C185R-RFNG (1.0 × 10^5^ cells/well) were seeded to cover glass coated with collagen type I-A (Nitta Gelatin) on 6-well plate and cultured for 24 h with and without 1 μg/ml dox. They were cocultured with JAG1-3T3 or MIG-3T3 (1.0 × 10^5^ cells/well) for 24 h and with DLL4-3T3 or MIG-3T3 (1.0 × 10^5^ cells/well) for 4 h. After the coculture, the following procedures were performed all at RT. After the coculture, the cells on the cover glass were fixed with 4% PFA for 20 min, permeabilized with 0.2% TritonX-100 in PBS for 5 min, blocked with 1% Block Ace (Pharma Biomedical) in PBS for 30 min, and then incubated with sheep anti-human NOTCH3ECD (R＆D system, AF1559, 1:400) in 0.4% Block Ace in PBS for 60 min. After the incubation, they were washed with PBST (0.1% Tween 20 in PBS) for 5 min three times and incubated with Rhodamine Red X-conjugated donkey anti-sheep IgG (H+L) (Jackson ImmunoResearch, 1:200∼1:400) in 0.4% Block Ace and 0.1% TritonX-100 in PBS for 30 min with shading. After the incubation, they were washed with PBST for 5 min three times and washed with MilliQ two times and then the cover glasses were fixed at slide glasses using Fluoromount (Diagnos). The obtained images using a fluorescence microscope (Keyence, BZ-X800; Evident, APX-100) were analyzed with Fiji (NIH). The total number of MIG-3T3, JAG1-3T3, and DLL4-3T3 contacting HeLa-N3WT/R141C/C185R-RFNG and those with N3ECD-positive vesicles were counted with Cell Counter, a plugin in imageJ (https://imagej.net/ij/) The percentages of N3ECD-positive MIG-3T3, JAG1-3T3, and DLL4-3T3 to the total were calculated.

As for Figure 7, N3WT/C185R-RF-HeLa cells were transfected with 10 pmol of NOTCH2 siRNA and precultured with or without 1 μg/ml doxycycline on cover glass coated with collagen type I-A (Nitta Gelatin) in a 6-well plate at a density of 0.7 × 10^5^ cells/well for 2 days (for Fig. 7). The cells were then cocultured with JAG1-3T3 cells (2.0 × 10^5^ cells/well) and treated with 200 μM leupeptin hemisulfate for 8 h. The cells were fixed with 4% PFA for 10 min, blocked with 1% Block Ace (Pharma Biomedical) for 30 min, and incubated with mouse anti-human N3ECD (Abnova, 1G5; 1:200) in 0.4% Block Ace for 60 min. After permeabilization with 0.2% Triton X-100 in PBS for 5 min, the cells were reblocked with 1% Block Ace and incubated with sheep anti-human N3ECD (R&D Systems, BAF1559; 1:400) in 0.4% Block Ace and 0.1% Triton X-100 for 60 min. Subsequently, the cells were washed with PBST (0.1% Tween 20 in PBS) for 5 min three times and incubated with Rhodamine Red X-conjugated donkey anti-sheep IgG (H+L) (1:500) and Alexa Fluor 647 AffiniPure Donkey Anti-Mouse IgG (H  + L) (Jackson ImmunoResearch; 1:500) in 0.4% Block Ace and 0.1% Triton X-100 for 60 min, with shading. After the incubation, the cells were washed with PBST for 5 min three times and washed with diluted water two times. Finally, the cover glasses were fixed on glass slides with Fluoromount (Diagnostic BioSystems). Confocal imaging was performed using a confocal microscope (SP8, Leica).

We quantified the percentage of JAG1-3T3 cells harboring NOTCH3 in the cell membrane using Fiji (NIH) software. We examined fluorescence with EGFP, rhodamine red, and Alexa Fluor 647 in the green, magenta, and cyan channels, respectively. After acquiring confocal z-stack images, we segmented the images into three channels. Binarization was applied to the magenta and cyan channels individually, and the merged signal was extracted by “Image calculator,” a plugin (hereafter, magenta + cyan). For both the magenta and magenta + cyan channels, we quantified the number of particles within the cellular region of JAG1-3T3, which was delineated in the green channel, using “Analyze Particles”, a plugin. After that, the particle number obtained from the magenta + cyan channel was divided by that obtained from the magenta channel, determining the ratio of NOTCH3 particles present in the cell membrane relative to the total particles per cell. Approximately 20 to 30 cells per sample were quantified for each experiment, and three independent experiments were conducted.

### PI staining

ci37/HBPC cells were cultured for 14 days in serum-free pericyte medium and washed with Hanks' balanced salt solution. Before and after fixation with PFA, the cells were stained by 500mM PI in saline-sodium citrate buffer for 5 min RT. Then, the cells were captured by APX100 (EVIDENT). N3WT/C185R-RF-HeLa were treated with 150 μg/ml puromycin in 35 mm dish for 16 h and stained by 500mM PI before fixation.

### Data presentation and statistical analysis

All figures display data from multiple independent biological samples, which are included in the Figure legend. The statistical significance of the differences was determined by tests, as shown in the Figure legend with GraphPad Prism 8 (GraphPad software, https://www.graphpad.com).

## Data availability

The glycoproteomics data from this publication have been deposited to the jPOST database (https://repository.jpostdb.org/) and assigned the identifier: JPST002465.

## Supporting information

This article contains supporting information.

## Conflict of interest

The authors declare that they have no conflicts of interest with the contents of this article.
