# Supplementary material for: The N-acetylglucosaminyltransferase Radical fringe contributes to defects in JAG1-dependent turnover and signaling of NOTCH3 CADASIL mutants
Source: J Biol Chem. 2024 Sep 19;300(10):107787. doi: 10.1016/j.jbc.2024.107787 (PMC11525139; doi:10.1016/j.jbc.2024.107787)
Supplement: Supporting Information [file mmc1.docx]

Supporting information (Figures S1-9, Tables S1-2)

Radical fringe contributes to defects in JAG1-dependent turnover and signaling of NOTCH3 CADASIL mutant proteins, R141C and C185R

Shodai Suzuki, Taiki Mashiko, Yohei Tsukamoto, Miyu Oya, Yuki Kotani, Saki Okawara, Takemi Matsumoto, Yuki Mizue, Hideyuki Takeuchi, Tetsuya Okajima, Motoyuki Itoh

CONTENT

1. Figure S1 …2
2. Figure S2 …3
3. Figure S3 …4
4. Figure S4 …5
5. Figure S5 …6
6. Figure S6 …7
7. Figure S7 …8
8. Figure S8 …9
9. Figure S9 …10
10. Table S1 …11
11. Table S2 …12

**
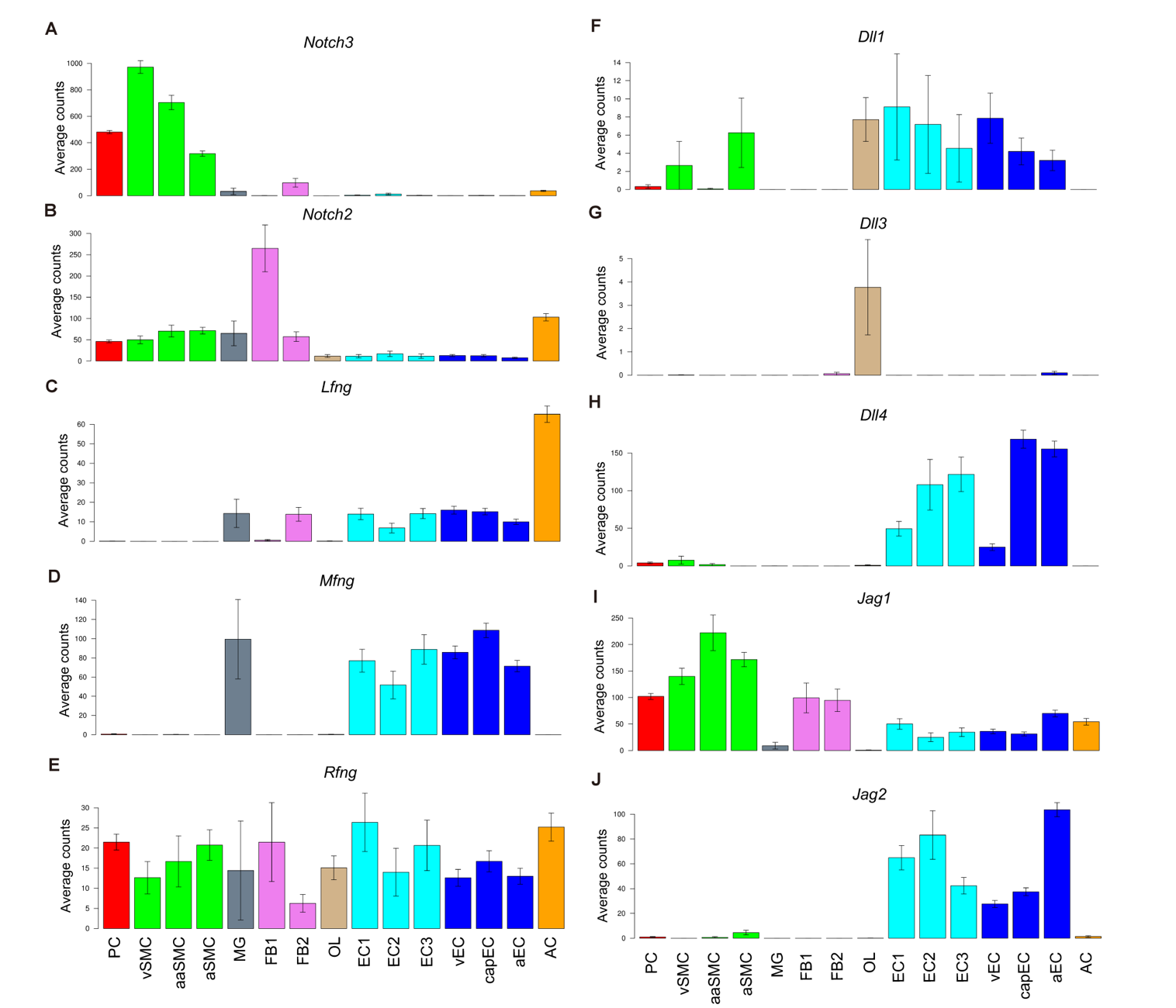
**

**Figure S1 - The mRNA levels of Notch3, Fringe genes, and Notch canonical ligands in mouse brain vascular cells**

*(A-J)* Read counts of *Notch3 (A), Notch2 (B), Lfng (C), Mfng (D), Rfng (E), Dll1 (F), Dll3 (G), Dll4 (H), Jag1 (I),* and *Jag2 (J)* in mouse adult cerebral vascular cells from the single-cell RNA-seq database (<http://betsholtzlab.org/VascularSingleCells/database.html>) (Vanlandewijck *et al*, 2018; He *et al*, 2018). vSMC, venous smooth muscle cell. aaSMC, arteriolar smooth muscle cell. aSMC, arterial smooth muscle cell. MG, microglia. FB1/2, fibroblast-like type 1/2. OL, oligodendrocyte. EC1/2/3, endothelial cell type 1/2/3. vEC, venous endothelial cell. capEC, capillary endothelial cell. aEC, arterial endothelial cell. AC, astrocyte.


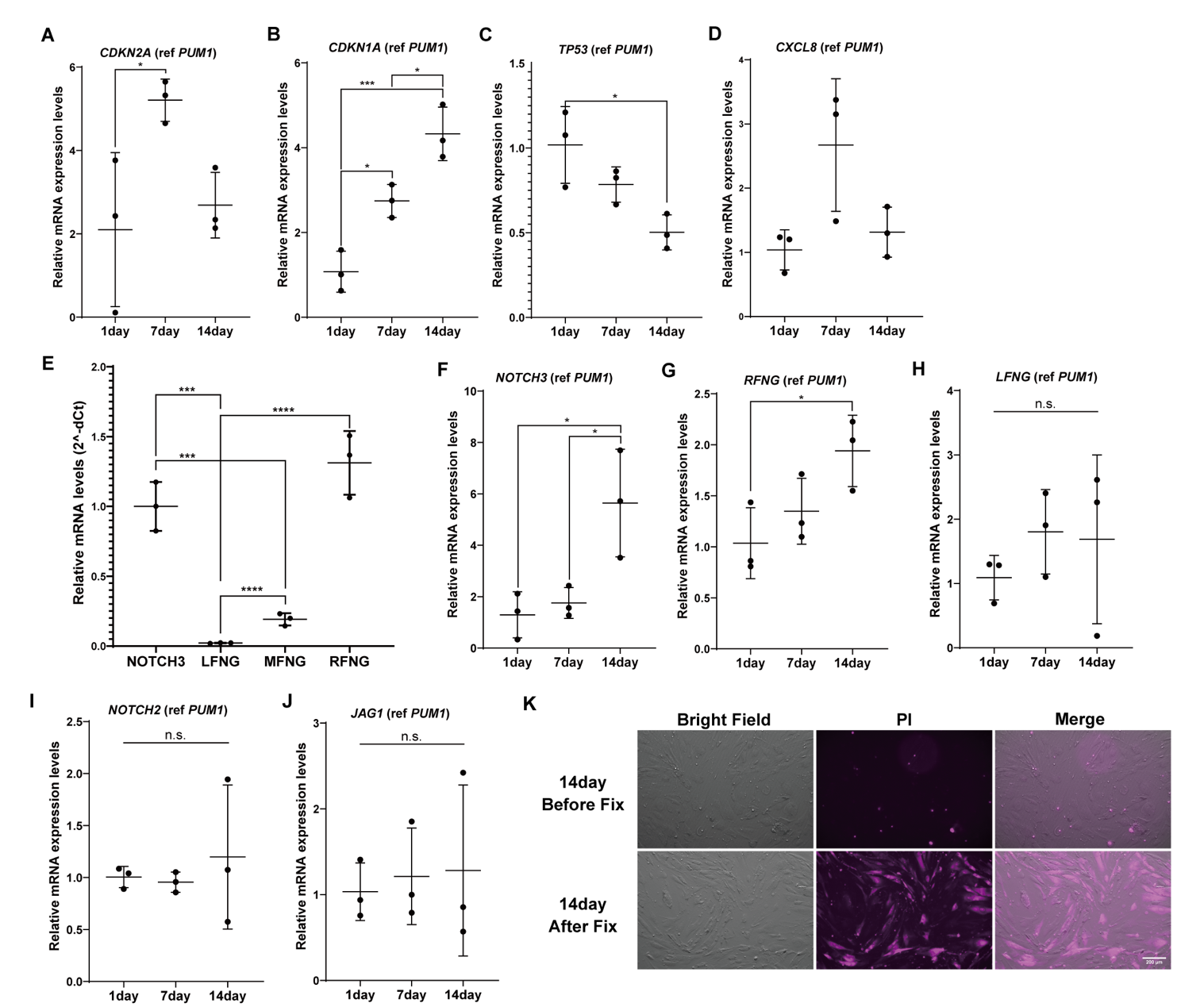


**Figure S2 – Senescence promotes the expression of the NOTCH3 and RFNG mRNAs in pericytes**

*(A-D)* qRT‒PCR showing the relative mRNA levels of *CDKN2A (A), CDKN1A (B), TP53 (C), and CXCL8 (D)* in HBPC/ci37 cells cultured for 1, 7, and 14 days (independent biological replicates N=3). The values were obtained by calculating the ΔΔCt of each gene normalized to the value of *PUM1*.

*(E)* qRT‒PCR showing the relative mRNA levels of NOTCH3 and Fringe homologs in HBPC/ci37 cells (independent biological replicates N=3). The values were obtained by calculating the ΔCt of Fringe relative to that of GAPDH and normalizing to that of NOTCH3.

*(F-J)* qRT‒PCR showing the relative mRNA levels of *NOTCH3 (F), RFNG (G), LFNG (H), NOTCH2 (I),* and *JAG1 (J)* in HBPC/ci37 cells cultured for 1, 7, and 14 days (independent biological replicates N=3).

*(K)* PI stained images of HBPC/ci37 cells cultured for 14 days before and after fixation with PFA. The left, middle, and right panels show blight field, PI, and overlays, respectively. Scale bar, 200μm.

Data information: In A-J, data are presented as mean ± SD. n.s., not significant, *p<0.05, ***p<0.001, ****p<0.0001 (Tukey’s *post hoc* test following one-way ANOVA).

**
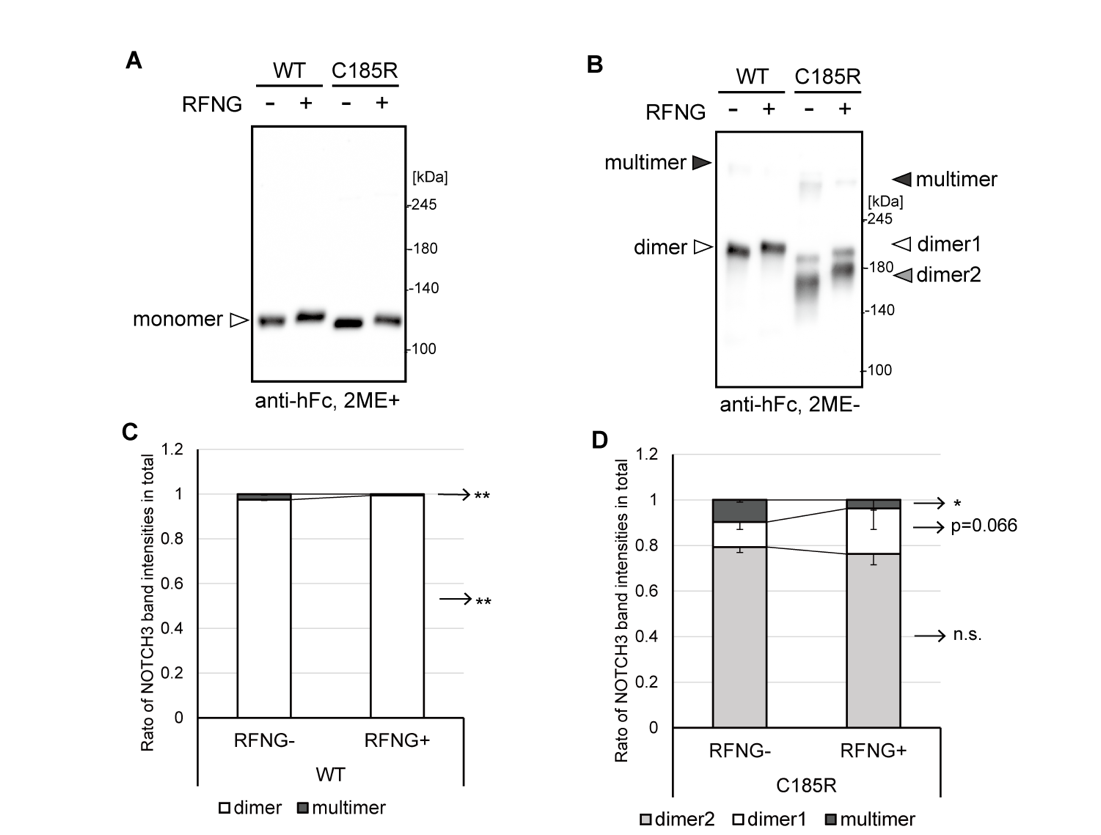
**

**Figure S3 – Non-reducing SDS-PAGE of NOTCH3 EGFr1-12 WT and C185R**

*(A, B)* Representative western blot of purified NOTCH3 EGFr1-12 WT and C185R-Fc-6×His, with or without RFNG-mediated modification, separated by SDS‒PAGE with (*A)* and without *(B)* 2-mercaptoethanol (2ME). NOTCH3 was detected by an antibody against human Fc (independent biological replicates N=3).

*(C, D)* Proportion of dimers and multimers in the NOTCH3 WT *(C)* and C185R *(D)* against the total, as determined by nonreducing SDS‒PAGE in *(B)* (independent biological replicates N=3)*.*

Data information: In C-D, data are presented as mean ± SDs. n.s., not significant (p>0.05); *p<0.05, **p<0.01 (Unpaired two-tailed Student’s t-test)

**
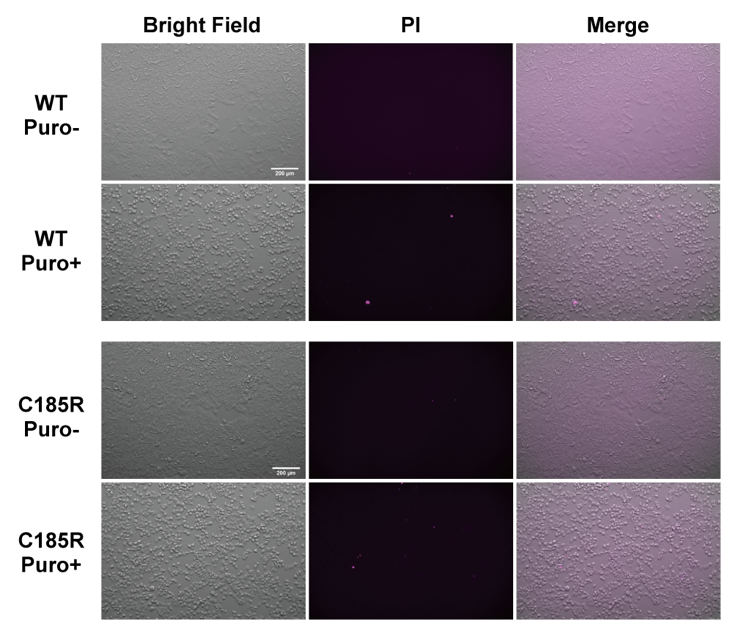
**

**Figure S4 – Cell viability of HeLa cell lines expressing NOTCH3 WT and C185R treated with puromycin**

PI staining images of N3WT/C185R-RF-HeLa treated with or without 150μM puromycin for 16 hours. Left, middle, and right panels show blight field, PI, and overlays, respectively. Scale bar, 200μm.


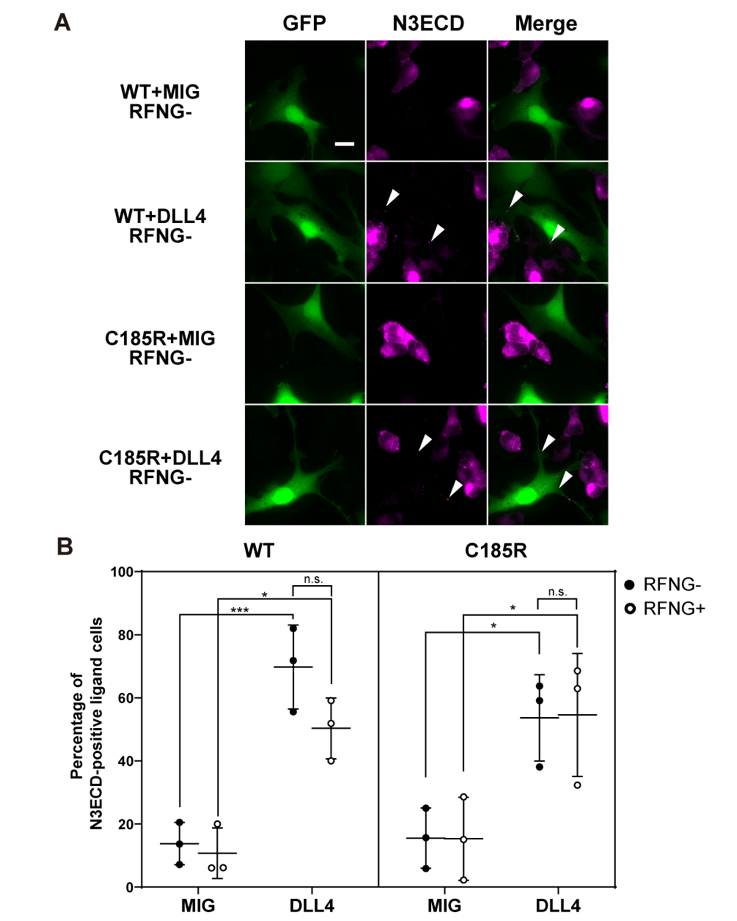


**Figure S5 - RFNG does not affects degradation of NOTCH3 WT and C185R endocytosed by DLL4**

*(A)* Representative images of immunocytochemistry with an N3ECD antibody after co-culturing N3WT/C185R-RF-HeLa with DLL4-3T3 cells. N3WT/C185R-RF-HeLa was cultured with and without dox for one day before co-culturing with MIG-3T3 or DLL4-3T3 for four hours. The left panel shows GFP expressed in MIG-3T3 and DLL4-3T3 (green). The middle panel shows N3ECD (magenta). The right panel shows an overlay. Arrows indicate N3ECD trans-endocytosed into DLL4-3T3. Scale bar, 10μm.

*(B)* Proportion of DLL4-3T3 and MIG-3T3 positive with NOTCH3 WT and C185R (independent biological replicates, N=3).

Data information: In B, data are presented as mean ± SD. *p<0.05, ***p<0.001, ****p<0.0001, n.s., not significant (Tukey’s *post hoc* test following three-way ANOVA).


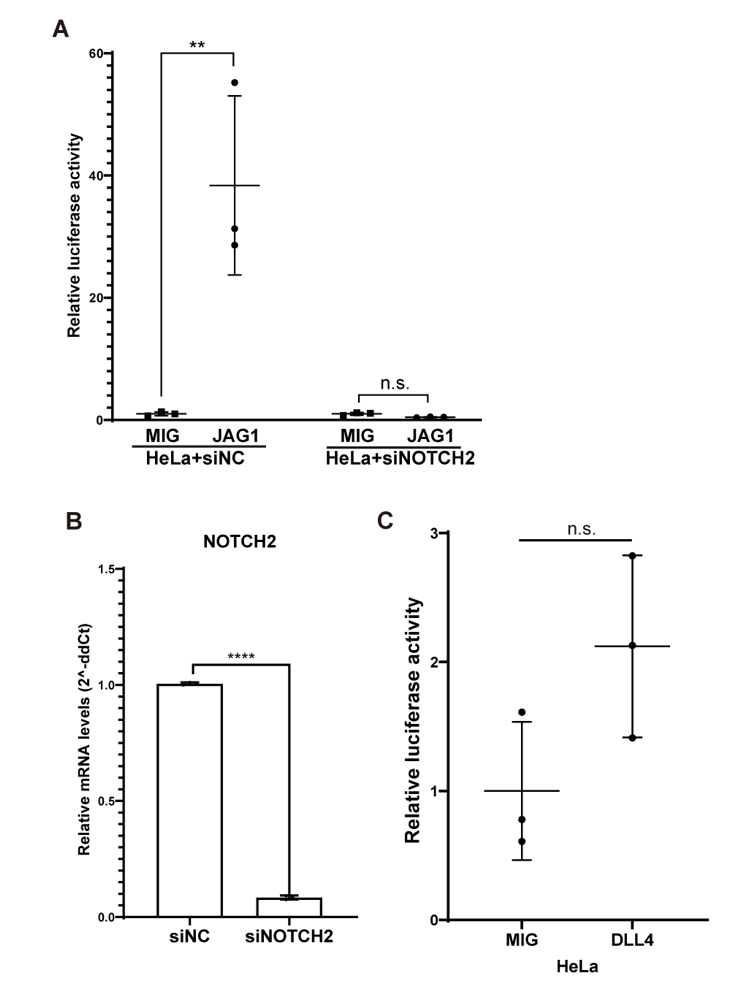


**Figure S6 - Endogenous Notch signaling activity in HeLa cells**

*(A)* Notch reporter assay with lysates collected from HeLa cells cocultured with JAG1-3T3 or MIG-3T3 cells with or without NOTCH2 expression (independent biological replicates N=3). The Notch signaling activity was calculated relative to that observed after coculture with MIG-3T3 cells.

*(B)* qRT‒PCR showing relative mRNA levels of *NOTCH2* in HeLa cells transfected with siNOTCH2 (independent biological replicates N=3). The values were obtained by the ΔΔCt method. GAPDH was used as a reference gene.

*(C)* Notch reporter assay with lysates collected by coculturing HeLa cells with DLL4-3T3 or MIG-3T3 (independent biological replicates N=3).

Data information: In A-C, data are presented as mean ± SD. n.s., not significant, *p<0.05, ***p<0.001, ****p<0.0001 (A:Tukey’s *post hoc* test following one-way ANOVA, B and C: Unpaired two-tailed Student’s t-test).


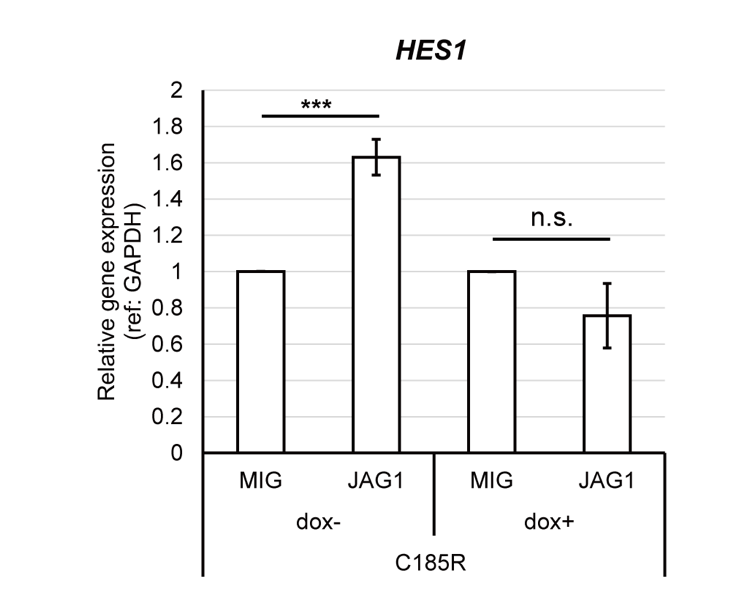


**Figure S7 – RFNG inhibits upregulation of HES1 by JAG1 in NOTCH3 C185R cells**

Relative mRNA levels of *HES1* after co-culturing N3WT/C185R-RF-HeLa with MIG-3T3 cells or JAG1-3T3 cells for 24 hours under treatment with siNOTCH2. The values were obtained by the ΔΔCt method of qRT-PCR. GAPDH was used as a reference gene (independent biological replicates N=3). Data are presented as mean ± SD. n.s., not significant, ***p<0.001 (Unpaired two-tailed Student’s test).


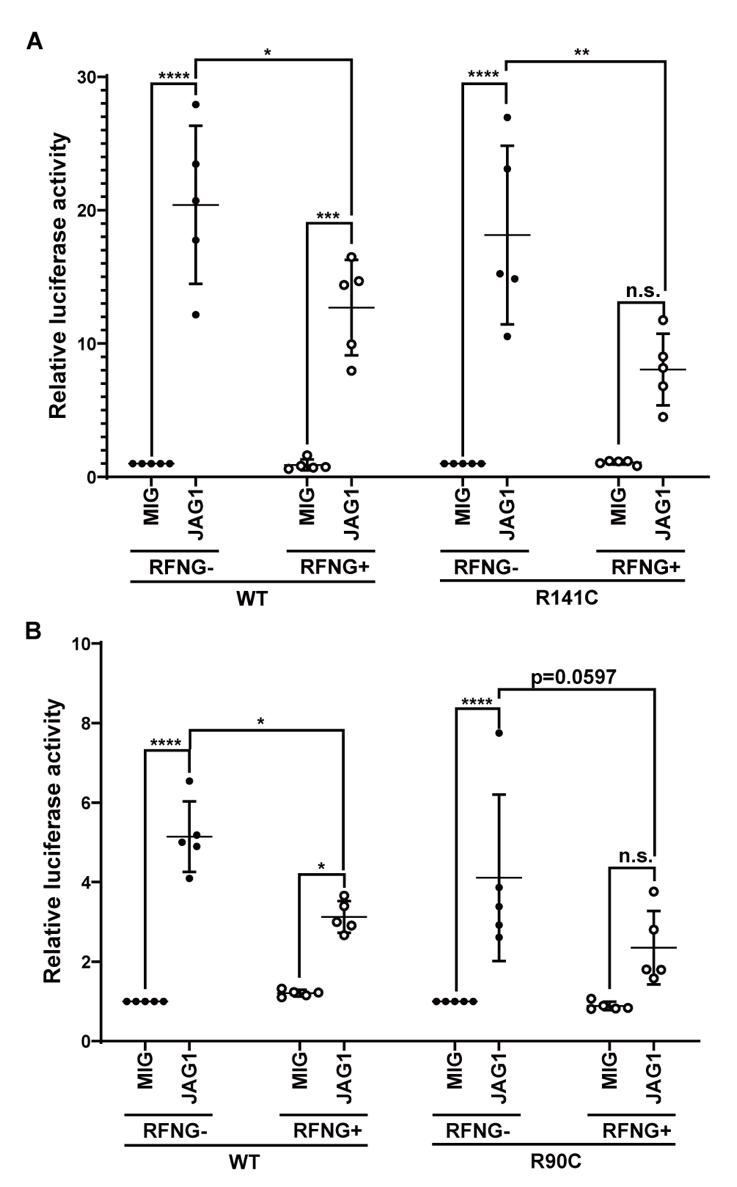


**Figure S8 – JAG1-dependent signal activities of NOTCH3 R90C and R141C**

*(A)* Notch reporter assay with the collected lysates from cocultures of N3WT/R141C-RF-HeLa and JAG1-3T3 (independent biological replicates N=5).

*(B)* Notch reporter assay with the collected lysates from cocultures of N3WT/R90C-RF-HeLa and JAG1-3T3 (independent biological replicates N=5).

Data are presented as mean ± SD. n.s., not significant, *p<0.05, **p<0.01, ***p<0.001, ****p<0.0001 (Tukey’s *post hoc* tests following three-way ANOVA).

**
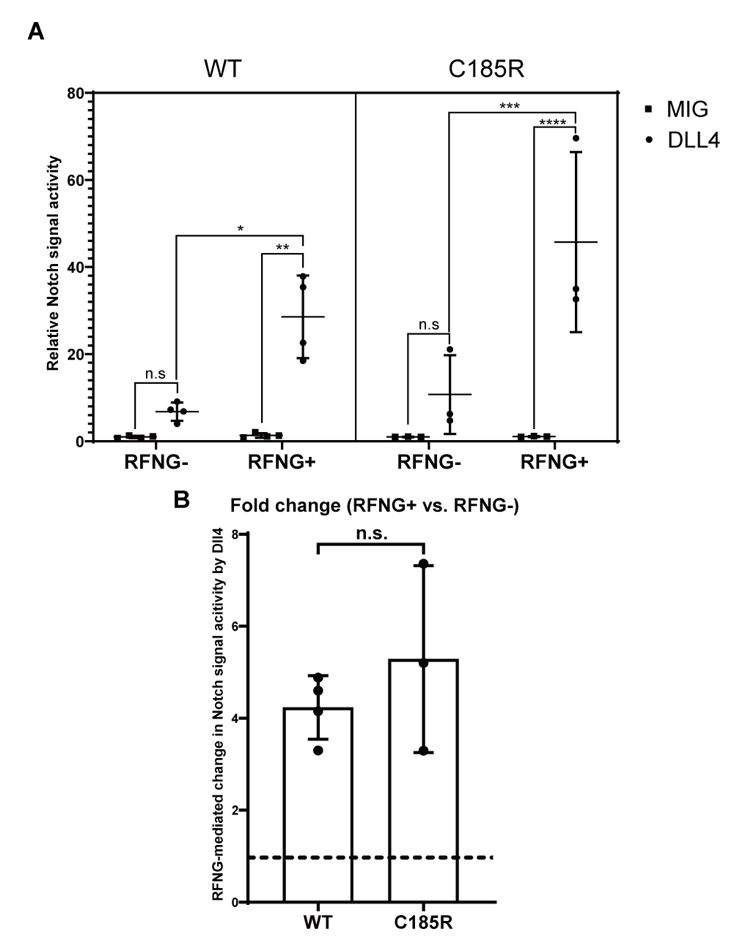
**

**Figure S9 - RFNG enhances the DLL4-mediated signaling activities of NOTCH3 WT and C185R**

*(A)* Notch reporter assay with lysates from cocultures of N3WT/C185R-RF-HeLa with DLL4-3T3 or MIG-3T3 cells. NOTCH3 signaling activity was calculated relative to that observed upon coculture with MIG-3T3 cells (independent biological replicates WT, N=4; C185R, N=3).

*(B)* Fold change in DLL4-mediated activities of NOTCH3 WT and C185R with RFNG coexpression against those without RFNG (independent biological replicates WT, N=4; C185R, N=3).

Data information: In A and B, data are presented as mean ± SD. n.s., not significant, *p<0.05, **p<0.01, ****p<0.0001 (A: Tukey’s *post hoc* test following two-way ANOVA; B: Unpaired two-tailed Student’s t-test)

| **Name** | **For/Rev** | **Sequence (5'→3')** |
| --- | --- | --- |
| RFNG202 | For | GGGAATTCACCGTGTCATCAACACCAACTGCTC |
|  | Rev | GGGCGGCCGCGTCTTCGTTCTCCCAAAACACCCATC |
| RFNG201  overlap-PCR-1  (RFNG208 region) | For | CGGGGACGACCCTGAGCTCGAGCTCCAGGGCGGCGACCGTGTCATCAACACCAACTGCTC |
|  | Rev | GCACCGCCGAGCAGTTGGTGTTGATGACACGGTCGCCGCCCTGGAGCTCGAGCTCAGGGT |
| RFNG201  overlap-PCR2 (RFNG202 region) | For | GGGTCGACGCCAAATCGGCCGAGCTCGAATTCGTC |
|  | Rev | CCGAATTCCTAAGCGTAATCTGGAACATCGTATGGGTACCGAGAGGTCGGGGCGCCCTGT |
| RFNG201  inverse-PCR | For | ATGATGACAATTATGTGAACGCCAGGAGCCTCCTGC |
|  | Rev | CCACGTGGCAAAACCACTTGCGCCCGGACTCAATG |
| NOTCH3 EGFr1-12 | For | ATCTCAGAAGAGGATCTGTCCGGAATGGGGCCGGGGGCCCGTGG |
|  | Rev | CTCAACAGATTGAGATCTTCCGGACTGACACGTGGAGCCGCTGAAG |

**Table S1 - PCR primer list for construction**

| **Gene** | **For/Rev** | **Sequence (5'→3')** |
| --- | --- | --- |
| GAPDH | For | GAAATCCCATCACCATCTTCCAGG |
|  | Rev | GAGCCCCAGCCTTCTCCATG |
| NOTCH2 | For | ATGACTGCCCTAACCACAGG |
|  | Rev | TGCAGTCATCTCCACTCCAG |
| NOTCH3 | For | GGCATCAACCGCTACGACT |
|  | Rev | CCATTTTCCCCATCCACAC |
| LFNG | For | TCCGGCAGGAAGTGGTTCT |
|  | Rev | TCCAGAGTGTGCGGGTAGCT |
| MFNG | For | AGCTACACGATGTCTTCATTGCA |
|  | Rev | CCTGGAAACCCACGTGTCA |
| RFNG | For | CCACGTGGATGATGACAATTATG |
|  | Rev | CCTGGCTGGGTGAGAAGCT |
| JAG1 | For | GCTTGTGGAGGCGTGGGATT |
|  | Rev | TTGATCATGCCCGAGTGAGAA |
| CDKN2A | For | GAGCAGCATGGAGCCTTC |
|  | Rev | CCTCCGACCGTAACTATTCG |
| CDKN1A | For | GACACCACTGGAGGGTGACT |
|  | Rev | CAGGTCCACATGGTCTTCCT |
| TP53 | For | AGGCGCTGCCCCCACCATGA |
|  | Rev | GGCGGCTCATAGGGCACCACCA |
| GUSB | For | AAGTCCTTCACCAGCAGCG |
|  | Rev | CCACGGTGTCAACAAGCAT |
| PUM1 | For | CGGTCGTCCTGAGGATAAAA |
|  | Rev | CGTACGTGAGGCGTGAGTAA |

**Table S2 - qPCR primer list**
